# Advances in Cereal Crop Genomics for Resilience under Climate Change

**DOI:** 10.3390/life11060502

**Published:** 2021-05-29

**Authors:** Tinashe Zenda, Songtao Liu, Anyi Dong, Huijun Duan

**Affiliations:** 1 State Key Laboratory of North China Crop Improvement and Regulation, Hebei Agricultural University, Baoding 071001, China; liusongtao@hebau.edu.cn (S.L.); donganyi@hebau.edu.cn (A.D.); 2 North China Key Laboratory for Crop Germplasm Resources of the Education Ministry, Hebei Agricultural University, Baoding 071001, China; 3 Department of Crop Genetics and Breeding, College of Agronomy, Hebei Agricultural University, Baoding 071001, China; 4 Department of Crop Science, Faculty of Agriculture and Environmental Science, Bindura University of Science Education, Bindura P. Bag 1020, Zimbabwe

**Keywords:** cereal crops, genome sequencing, mutagenesis, gene editing technologies, long non-coding RNAs (lncRNAs), crop phenotyping, crop wild relatives, pan-genomes

## Abstract

Adapting to climate change, providing sufficient human food and nutritional needs, and securing sufficient energy supplies will call for a radical transformation from the current conventional adaptation approaches to more broad-based and transformative alternatives. This entails diversifying the agricultural system and boosting productivity of major cereal crops through development of climate-resilient cultivars that can sustainably maintain higher yields under climate change conditions, expanding our focus to crop wild relatives, and better exploitation of underutilized crop species. This is facilitated by the recent developments in plant genomics, such as advances in genome sequencing, assembly, and annotation, as well as gene editing technologies, which have increased the availability of high-quality reference genomes for various model and non-model plant species. This has necessitated genomics-assisted breeding of crops, including underutilized species, consequently broadening genetic variation of the available germplasm; improving the discovery of novel alleles controlling important agronomic traits; and enhancing creation of new crop cultivars with improved tolerance to biotic and abiotic stresses and superior nutritive quality. Here, therefore, we summarize these recent developments in plant genomics and their application, with particular reference to cereal crops (including underutilized species). Particularly, we discuss genome sequencing approaches, quantitative trait loci (QTL) mapping and genome-wide association (GWAS) studies, directed mutagenesis, plant non-coding RNAs, precise gene editing technologies such as CRISPR-Cas9, and complementation of crop genotyping by crop phenotyping. We then conclude by providing an outlook that, as we step into the future, high-throughput phenotyping, pan-genomics, transposable elements analysis, and machine learning hold much promise for crop improvements related to climate resilience and nutritional superiority.

## 1. Introduction

Combating global climate change, providing sufficient human nutritional needs, and securing sufficient energy supplies are the formidable challenges confronting humankind in the current era [1,2,3,4]. Cereal crops, which are characteristically grasses cultivated for their edible grains [5], are the major suppliers of food energy to human beings and livestock and hence are produced in greater quantities more than any other crop species [6]. Among cereal crops, wheat (*Triticum aestivum* L.), rice (*Oryza sativa* L.), and maize (*Zea mays* L.) constitute the top three most important, in terms of production [6,7,8,9]. Nearly 40% of our daily calories are dependent on those three cereals which require more resources than climate-smart ‘minor’ cereal crops such as pearl millet (*Pennisetum glaucum* L.) [10]. Other cereal crops include barley (*Hordeum vulgare* L.), oat (*Avena sativa* L.), rye (*Secale cereal* L.), sorghum (*Sorghum bicolor* L.), pearl millet, foxtail millet (*Setaria italica* L.), finger millet (*Eleusine coracana* L.), barnyard millet (*Echinochloa esculenta* L.), fonio millet (*Digitaria exilis* L.), and tef (*Eragrotis tef* L.), among others [11,12]. However, the continued production of major cereal crops, particularly the top three cereals, is endangered by climate change, consequently threatening global food security [13,14,15,16,17,18].

In the past century, agriculture has succeeded in feeding the world population through variety crossing and selection by breeders to enhance characters of cultivated crops and innovative agronomic interventions of the 1960s which encompassed greater use of mineral fertilizers and irrigation [19,20,21]. This brought about momentous crop yield gains which resulted in significant food production surges in cereal crops, particularly maize, wheat, and rice [20,22]. However, the contemporary homogenized food production system that is characterized by resource-intensive crop species possessing high calories quantities, but limited nutritional diversity, has reduced genetic diversity in most cultivated crops, consequently limiting their climate adaptation capacity, as well as contributing to malnutrition issues [23,24,25,26,27]. More frightening is the realization that the higher yield gains of the last few decades are now difficult to sustain, largely due to ‘elite’ crop varieties reaching their full genetic potential, eroding genetic diversity, dwindling arable land for agriculture as a result of urbanization and land degradation, and climate change [22,28,29,30].

Diversifying the global food supply, boosting agricultural productivity and tackling nutritional challenges, whilst adapting to climate change, would call for a radical transformation from the current conventional adaptation approaches to more broad-based and transformative alternatives [31]. This entails developing climate resilient crop cultivars that can sustainably maintain their productivity under such climate-change exacerbated conditions, expanding our focus to crop wild relatives (CWRs) and better exploitation of underutilized crop species (UCS) [23,27,31,32,33,34]. This is facilitated by the recent advances in plant genomics, which have revolutionized our capacity to mine key genes regulating critical agronomic traits [2,20].

Genomics, which is the investigation of the complete DNA sequence of a particular organism [35], in plants involves the study of all features of plant genomes and individual specific genes at the DNA level, including different types of mutations, polymorphisms, and sequence-difference-based phylogenetic connections [36]. It comprises structural genomics, functional genomics, and comparative genomics [37,38,39]. Structural genomics covers genome structure characterization, particularly aiming at identification and understanding of the physical architecture of the genome, and location and identification of genomic features of the chromosomes; this is essential for gene or DNA manipulation efforts aimed at creating valuable agronomic traits [12,38]. On the other hand, functional genomics involves describing the gene function using voluminous information encompassing sequence and mapping data, as well as gene function characterization [12]. For instance, transcriptomics constitute functional genomics, which are concerned with both qualitative and quantitative patterns of transcription [36]. In the field of crop science, comparative functional genomics and transcriptomics are primary concerned with the identification of allelic variations governing the improved phenotype [39]. For a crop species, its genomic resources encompass its genome sequence, gene functional annotation, and existing diversity in the gene pool [3]. A combination of all these genomic tools aids in developing variant panels such as single nucleotide polymorphisms (SNPs), which are essential for associating and introgressing important plant agronomic and economic traits [3]. A wide array of functional genomics tools has been employed to understand plant gene functions and their related governing networks [38,40,41], and functional genomics studies may offer the most accessible and useable data for crop improvement programs [12].

Genomic technologies, applied together with new methods like gene editing and rapid generation turnover such as genomic selection (GS), have the capacity to accelerate the rate of genetic gains in crop breeding programs [42]. Therefore, harnessing these technologies will lessen the breeding cycles and reduce breeding costs whilst improving crop traits for adaptation to climate change and enhancing nutritional value [39].

With regards to crop improvement efforts, climate change is compelling plant breeders to explore useful traits from all possible sources [43]. Therefore, harnessing UCSs in crop breeding programs helps breeders in accessing diversity for specific traits of importance that are harbored in such orphaned crop species but absent in elite crop genotypes [26]. Notwithstanding their proven economic and agro-ecosystem significance, a lag in the overall genetic improvement and up-scaling of orphan crops still persist [11,32,44]. However, the need to adapt to climate change and address food and malnutrition challenges through nutritious, sustainable, and resilient agri-food systems, as well as the advancement of genomic technologies and gene mapping tools such as precise genome editing using the clustered regularly interspaced short palindromic repeats (CRISPR) and CRISPR-associated protein 9 (Cas9) (CRISPR-Cas9) system and genome-wide association study (GWAS), have renewed the interest in orphaned and underutilized crop species [3,33,45]. Particularly in low-income countries, these orphaned crops offer vital opportunities to improve nutritional quality and sustainability of food production system, since they are nutrients-dense, can properly fit into multiple production system niches, and relatively adapt to low-input environments [25,46].

Here, we summarize the recent developments in plant genomics and their application, with particular reference to cereal crops (including millets as underutilized species). In particular, we discuss DNA sequencing platforms, quantitative trait loci (QTL) mapping and GWAS studies, mutagenesis, plant noncoding RNAs, precise gene editing technologies such as CRISPR-Cas9, and complementation of crop genotyping by crop phenotyping. We then conclude by giving an outlook of future plant genomics research with regards to crop climate resilience and nutritional superiority.

## 2. De Novo Domestication of Crop Wild Relatives and Better Exploitation of Orphan Crop Species

Development of new germplasm resources with novel allelic diversity in useful backgrounds is critical in assisting the identification of genes that contribute to important adaptive traits [26]. De novo domestication of CWRs becomes central if crop breeders are to create new varieties with novel climate adaptive traits. CWRs refer to plant species sharing close phylogenetic resemblance with domesticated crops, from any geographical location in the world. Examples of CWRs include landraces, crop progenitors, and some closely related plant taxa without known agriculture significance [47,48]. Long periods of evolution and selective pressure (natural selection) have allowed CWRs to accumulate several important genes enabling them to survive harsh biotic and abiotic environments [20]. Compared with modern, elite cultivars, which are normally selected for high-input environments with minimal limitations, CWRs exhibit morpho-physiological features for survival and adaptation under extreme conditions [49]. Therefore, CWRs and landraces serve as potential reservoirs of beneficial alleles for tolerance to abiotic stresses such as drought, heat, salt, and cold, that can be introduced to crop lines via traditional or molecular breeding [26,50,51,52]. For instance, grain sorghum can be improved by exploitation of the yet untapped potential of the extensive gene pool of CWR in its genus [47]. *Sorghum* is a genus within the tribe Andropogoneae that includes other plant genera such as *Saccharum* (sugarcane) and *Miscanthus* that are important biomass crops. Sugarcane and sorghum are more phylogenetically connected and have been observed to be inter-crossed [53,54]. Therefore, CWR in the *Sorghum* genus may be a valuable genetic resource for new crop development across the tribe, either via incorporation of important genes into genera such as *Saccharum* or by domestication of additional *Sorghum* species [35,47,55]. Additionally, cultivated barley (*Hordeum vulgare* L.) can be enhanced for drought tolerance by crossing it with its wild relative *Hordeum spontaneum* L. which harbors alleles for drought tolerance [20,48,49]. For cultivated maize, enhancement can be achieved by exploiting its wild relatives (called teosinte) such as *Zea parviglumis* (teosinte) and *Tripsacum* [20,56,57,58,59]. Interestingly, new advances in gene editing technologies such as CRISPR-Cas9 system may aid in bridging the strong reproductive and genetic hurdles in gene transfer between cultivated crop species and CWRs [47].

Interestingly, the utility of CWRs to unlock marginalized areas for agriculture has been gathering attention in recent years [60]. Moreover, the current technological advances in plant genomics and genome editing offer a window for accelerated domestication of CWRs and improvement of neglected, semi-domesticated crop species by targeting a few key genes and metabolic pathways [42,60]. Genomics have the capacity to expand the diversity of alleles in crop breeders’ toolkit by digging into the gene pools of CWRs [23]. Genomics may be used to characterize CWR populations and help their conservation and utilization or can be used to create CWR reference genomes that are useful in comparative genomics analyses [20,50]. However, CWR materials present challenges to crop breeders by requiring genetic selection, to become useful tools for agronomic and breeding programs, through pre-breeding [26].

The orphan crops are envisaged as the future climate-smart crops and are now gaining global recognition [44]. Such UCSs refer to neglected species cultivated mainly in their centers of origin or centers of diversity by native inhabitants where they are central to sustenance of local communities by providing special ecological, production, and consumption related roles, and underutilized species that were once widely grown but now degraded to disuse because of a range of agronomic, genetic, and economic reasons [61,62,63,64,65]. Nevertheless, these crops are nutrient dense [31,34,66,67] and highly adapted to marginal and complex environments, have contributed immensely to diversification and resilience of agroecological niches [4,65,68], and already occupy special positions with regards to the region’s socio-economic status and local farmers or consumers’ preferences [32]. These crops include millets (pearl, foxtail, finger, fonio, barnyard, etc.), tef, and *Amaranthus hypochondriacus* L., a pseudo-cereal crop, among others [12,34,44].

Millets can be used as valuable genetic breeding tools. For instance, foxtail millet harbors novel genes, alleles, and QTL for genetic improvement of major cereal crops and bioenergy grasses [23,69]. Moreover, pearl millet is a climate-smart crop, containing superior nutritive value (with greater amounts of zinc and iron) than wheat and other major cereals on top of being more resilient to climate stressors [27,46,70,71,72]. It is an alternative to major cereal crops because it can provide nutrition without the need of too much water and has a much greater resilience to heat and drought compared to wheat, rice and maize [10,27]. The climate-adaptive reproductive, phenotypic, and physiological characteristics of pearl millet give it thriving ability to grow in marginal conditions characterized by limited soil water availability, poor soil fertility, high salt content, high temperatures, and scant rainfall, where major cereal crops perform dismally [72]. Fortunately, pearl millet genome has been sequenced [73]. Other orphan cereal crops that have been whole-genome sequenced include foxtail millet [74,75], tef [32], fonio millet [60], and finger millet, among others [34,44]. Therefore, harnessing CWRs and better utilization of these UCSs will increase our genetic resource base and diversify our available gene pools since these species have been identified as sources of novel abiotic-related and nutrition-related traits that can be incorporated into major cereal crops [2,27,44,50,72,73]. However, to achieve global food security, research programs and political efforts will be necessary to make these UCSs available compared to a sole focus on only a few major staple food crops [10].

## 3. Advances in DNA Sequencing Technologies Accelerating Traits Discovery and Decoding Crop Species’ Whole Genomes

Reference genome sequences, as the basis of crop genetic and genomic studies, provide insights into gene content, genomic variation, and genetic foundation for agronomic traits [2,76]. High quality reference genome assemblies are important in elucidating complex traits and fast-tracking crop improvement by facilitating easy identification of favorable genes harboring better agronomic traits [77,78]. Particularly, whole genome sequences lay bare detailed genomic features, encompassing coding and noncoding genes, repetitive elements, GC content, and regulatory sequences that are valuable resources for deciphering plant genes’ functional roles [12].

The ground-breaking work of Sanger and his co-workers [79,80] initiated sequencing of DNA and genomes [12]. Particularly for the plant science, construction of the first complete genome sequence for *Arabidopsis thaliana* L. (Arabidopsis) in the year 2000 ushered in major strides for plant functional and comparative genomics studies [78,81]. Crop genomes of several major crops such as rice [82], maize [83], and sorghum [84] got decoded by the bacterial artificial chromosome (BAC)-based physical maps of the Sanger strategy [35,78]. The BAC physical maps used in Sanger sequencing offered a good template for completing gaps and errors, although the genome coverage of physical maps was sometimes non-representative due to cloning bias [35]. Thus, although the Sanger sequencing technology boasted of long read length and high assembly accuracy [85] and enabled the construction of ‘standard’ reference genomes for maize, rice, sorghum, and Arabidopsis [78], its widespread adoption suffered from its low throughput capacity and high cost of acquisition and operation [2].

However, post 2010, there has been tremendous progress in whole genome sequencing of several plant species, including CWRs and orphan crops (Table 1, [33,50,86,87,88]). Crucially, the evolution of sequencing platforms has allowed the generation of large volumes of sequencing data within a short period of time, at reduced costs as compared to the first-generation sequencing technologies [12]. In particular, the rapid development of second-generation or next-generation sequencing (NGS) technologies around the year 2010 onwards has facilitated assemblage of hundreds of plant genomes (Table 1, [20,86]). Prominent among the NGS technologies has been the Illumina platform, with its high throughput (HTP) and lower cost [89].

With the aid of NGS technologies, re-sequencing of the plant genome and the whole transcriptome in greater depth has been made possible [35,90,91]. For example, McCormick et al. [90] used deep whole-genome sequencing, coupled with high-density genetic map and transcriptome data to update the sorghum reference genome sequence (ver. 1) and its annotation as well as characterize additional features of the sorghum reference genome. They produced a resequenced high-quality sorghum reference genome (ver. 3) with improved sequence coverage (of ~29.6 Mb additional sequence), increased number of annotated genes (24% increase) to 34,211, increased average gene length, and narrowed error frequency rate by ten-fold (down to ~1 per 100 kbp) [90]. Moreover, sequencing of hundreds of related genomes within and between germplasm pools has facilitated deciphering of genetic diversity [12,47]. Meanwhile, NGS technologies possess some of the drawbacks of short read lengths, which limit their ability to span over long bits of repetitive sequences, causing misassemblies in the long repetitive regions and gaps in assemblies [2,20].

**Table 1:** Statistics of selected cereal crop species genome assemblies and annotation.

Species Name	Ploid Level	Genome Size	Assembled Genome (%)	^1^ Repeat Elements (%)	GC %	Genes	^2^ Sequencing Strategy	^3^ Public Year	References
*Triticum aestivum*	2n = 6x = 42 (AABBDD) allopolyploid	~17 Gb	14.5 Gb (85.29)	85.00	48.25	107,891	De novo WGS + BAC assemblies	2018	[12,86,92]
^4^ *Triticum urartu*	2n = 6x = 42 (AABBDD)	4.94 Gb	3.92 Gb (79.35)	66.88	46.00	34,879	WGS + Illumina	2013	[12,93]
*Oryza sativa*	2n = 2x = 24	389 Mb	370 Mb (95.12)	~51.00	~43.58–43.73	35,679	BAC PMs + Sanger seq.	2005	[12,35,82]
*Zea mays*	2n = 2x = 20	2.3 Gb	2.048 Gb (89.04)	85.00	46.91	47,800	BAC PMs + BAC seq.	2009	[35,83,86]
*Secale cereale*	2n = 2x = 14, RR	7.86 Gb	7.74 Gb (98.47)	90.31	45.89	45,596	PacBio + short read Illumina + Hi-C + Bio-Nano	2021	[94]
*Pearl millet*	2n = 2x = 14	~1.79 Gb	1.76 Gb (98.32)	68.16	47.90	38,579	WGS + BAC	2017	[73]
*Sorghum bicolor* (v1)	2n = 2x = 20	~730 Mb	625.6 Mb (85.70)	~63.00	44.50	~27,640	WGS + BACs + Sanger	2009	[35,84]
*Sorghum bicolor* (v3)	2n = 2x = 20	~700 Mb	655.2 Mb (93.60)	62.70	44.50	34,211	Deep WG short read seq. + Sanger + BAC PMs	2018	[90]
*Eleusine coracana*	2n = 4x = 36 (AABB)	1.45 Gb	1.19 Gb (82.31)	49.92	44.80	85,243	WGS + Illumina paired-end	2017	[12,95]
*Hordeum vulgare*	2n = 2x = 14	5.1 Gb	4.56 Gb (89.41)	~84.00	44.40	26,159	WGS	2012	[35,96]
*Setaria italica*	2n = 2x = 18	~490 Mb	~423 Mb (86.33)	~46.30	46.17	38,801	WGS + NGS	2012	[12,75]
*Setaria italica*	2n = 2x = 18	~510 Mb	~400 Mb (78.43)	~40.00	46.17	24,000– 29,000	WGS + Sanger + Illumina + BAC end seq.	2012	[12,35,74]
*Eragrotis tef*	2C = 2n = 4x = 40	772 Mb	672 Mb (87.05)	22.40	45.50	28,113	Illumina HighSeq 2000 single and paired-end	2014	[12,32]
*Digitaria exilis*	2n = 4x = 36	701.66 Mb	655.72 Mb (91.5)	49.00	-	57,021	Deep seq. of short reads + Illumina paired-end + Hi-C + Bionano optical map	2020	[32,97]

^1^ Repeat elements, refers to the portion of non-protein coding regions of the genome, comprising of transposons (*Activator*/*Dissociations (Ac/Ds)*, *Enhancer*/*Suppressor of mutation* (*En*/*Spm)*, *mutator* (*Mu*) elements, etc.), retrotransposons (long terminal repeats, LTRs; miniature inverted repeat TEs, MITEs; short interspersed nuclear element, SINE; long interspersed nuclear element, LINE, etc.), helitrons, etc. ^2^ WGS, whole-genome shotgun sequencing; BAC, bacterial artificial chromosome; BAC PMs, BAC physical maps; BAC seq., BAC sequencing; Hi-C, chromatin conformation capture; PacBio, Pacific Biosciences sequencing. ^3^ Public year, publication year of the genome sequence information. ^4^ The wheat specie *Triticum urartu* L. (einkorn wheat) is the progenitor species of the A genome. It is diploid wild wheat which resembles cultivated bread wheat (AABBDD) more extensively than any other wheat species.

Encouragingly, the emergence of third generation sequencing (TGS) approaches and generation of long-reads through platforms such as the Oxford Nanopore MinION (Nanopore) and Pacific Biosciences (PacBio) have offered the best way to resolve transposon repeats by generating long reads that span-over transposon regions, enabling distinct contiguous sequences to bridge-over the unknown locations, thereby facilitating the production of high-quality assemblies for complex genomes [78]. The PacBio and Nanopore long read sequencing approaches are single molecule real time (SMRT) methods, producing long reads (in real time) of several thousand bases which can span complex and repetitive regions [2]. Such TGS technologies can yield single molecule reads of ~60–200 Kb long, with an average length of 10–20 Kb [78,98]. However, these TGS approaches suffer from higher error rates (of approximately 13–18%) [81,99]. Various genome sequencing technologies across generations have been reviewed and compared in several papers [78,100,101]. The long-read sequencing technologies are usually coupled with optical mapping and confirmation capture to generate draft genomes of unparalleled contiguity [20,102]. Overall, the introduction of long-read sequencing approaches has presented a window for de novo assembling (scaffolding) of genomes, resolving sequence assembly ambiguities and gap filling. Moreover, the enhanced genome assembly improves spanning of regulatory sequences, consequently raising annotation efficiency and our capacity to identify functional genetic variations [78,103]. Further, this progress in genome sequencing has facilitated comparisons between related species and identification of subtle genetic variations that may be key in improvement of elite crops. For instance, PacBio long read single nucleotide sequencing strategy has been successfully used to explore the subtle genomic variations between sweet and grain sorghum reference genomes, where it was observed that among sucrose metabolism related genes, three sucrose transporters were either entirely eliminated or severely curtailed in the sweet sorghum variety Rio. However, several other sucrose transporters and sucrose synthases showed differential expression between the sweet and grain [91].

## 4. Approaches in Mapping of Genomic Regions Controlling Variation of Quantitatively Inherited Traits

Crop improvement largely depends on the availability and identification of genetic variation for the target traits and their utilization via breeding and transformation [35]. Meanwhile, the underlying gene regulatory processes governing crop biotic and abiotic stress responses are quite intricate, with several gene networks and stress signaling pathways being involved, and morpho-physiological traits affecting these crop responses are quantitatively inherited [104]. Beneficial alleles for various traits are located at specific chromosomal positions called QTL [49]. Therefore, the precise discovery of QTLs plays a crucial role in crop improvement through their manipulation via marker assisted selection (MAS) [105,106]. Fortunately, innovations in genomics-based methods offer access to these agronomically desirable alleles present at QTLs, and analysis of genome sequencing data and gene products facilitates the identification and cloning of genes at target QTLs [49]. For example, in maize crop improvement for drought tolerance, MAS has been employed to introgress QTL alleles for shortening the anthesis-silking interval [107].

Generally, the allelic variations of QTLs can be statistically linked with the value of a quantitative trait in two ways: across mapping populations (QTL or linkage mapping) or suitable panels of accessions characterized by the presence of linkage disequilibrium (LD)/association mapping) [49,108]. QTL or linkage mapping approach is the more traditional method and has been widely applied to identify genomic regions (QTL) controlling target traits [2,108,109]. This family-based mapping analysis relies on the genetic recombination and segregation during the construction of mapping populations in the progenies of bi-parental crosses that eventually affect the genetic mapping resolution and allele richness [104,109]. QTL mapping has demonstrated and remains a powerful tool to identify loci that co-segregate with the trait of interest in the research population. The utility of QTL mapping is that it can be applied in different population types such as F2 populations, double-haploid populations, backcross or recombinant inbred lines families, using different types of molecular markers [104,110]. Analysis of QTLs has identified several climate-related and nutrient-related QTLs in major cereal crops as extensively reviewed [23,39,109,110,111,112].

Worryingly, compared to other crops, research in millets is still lagging behind [46,113]. This is despite the fact that millets are considered predominantly climate resilient crops [11,34,72] and could serve as valuable source of novel genes, alleles, and QTLs for tolerance to climate-change-induced abiotic stresses [11,23]. Moreover, despite the high number of studies on QTL mapping for complex traits such as drought tolerance in major cereal crops over the past decade, there has been little success in introgression of those QTLs, and the number of causal genes that have been confirmed within these QTL regions remains relatively small as compared to Arabidopsis and rice [114,115]. Therefore, the identification and functional characterization of those stress-tolerance genes, alleles, and QTLs in millets is critical for their introgression and improvement of climate change resilience in cereal crops [23]. Promisingly, it is envisaged that within the next decade, necessitated by rapid improvements in high-throughput genome sequencing, crop phenotyping, and gene transfer techniques, QTL cloning will increasingly become feasible, whereas MAS will remain a useful tool for major QTL screening [23,72,116]. Cloned QTL facilitate a more targeted search for novel alleles and will offer novel insights for genetic engineering of climate resilient cereal crops [109].

Recent advances in NGS have enabled identification of major QTLs regulating specific plant phenotypes, via the development and deployment of enormous amounts of genetic markers such as single nucleotide polymorphisms (SNPs) and insertion-deletions (InDels), thereby aiding in an efficient way to enhance crop agronomic traits of economic importance, including in orphan crops. These developments in NGS facilitate the discovery of novel alleles/genes for various agronomic traits by genotyping-by-sequencing (GBS) approach [23]. The greater abundance of SNPs means that they cover a greater number of loci; hence, they are located in huge pools across the genome and can be used to classify sets of polymorphic markers [12]. Additionally, SNPs are amenable to high-throughput and automated profiling [49], therefore allowing for quick and high-throughput high-density SNP-marker-based genotyping [12,114]. Resultantly, SNPs have now overtaken other genetic markers such as single sequence repeats (SSR) markers as the preferred markers for marker assisted breeding, GWAS, GS, identification of disease-related alleles, or map-based cloning [12,114,117].

Genome-wide distributed high-density SNPs have greatly supported GWAS in delineating the slightest possible genome region associated with phenotypic variation in wide germplasm pools [42]. As a result, in the last few years, large-scale GWAS has become a key approach for mapping quantitative traits and studying the natural variation; GWAS is a powerful tool for performing effective and efficient genome-phenotype association analysis and identification of causative loci/genes for quantitative traits [81,104]. GWAS analysis approach involves evaluating statistical associations between DNA polymorphisms and trait variations across distantly related and heterogeneous individuals from a diverse collection that are genotyped and phenotyped for traits of interest [23,118]. Through screening large and diverse collections with ample genetic marker density, GWAS can effectively detect causal loci/gene underlying natural phenotypic variation; GWAS approach boasts robustness, high resolution, and effectiveness in the dissection of complex traits in crops [104]. Coupled with improved genome sequencing technology, GWAS enhances the mapping resolution for accurate location of allele/QTL/genes [23]. GWAS incorporates past recombination events in diverse association panels, and larger allele numbers, to identify genes linked to phenotypic traits at higher resolution than QTL analysis [2,104,108,119]. An increasing number of papers highlighting the use of SNPs in GWAS to detect genomic regions and candidate genes for various agronomic traits, including abiotic stress tolerance, in cereal crops are available for rice [120,121,122], pearl millet [72], barley [104,123], foxtail millet [124], sorghum [125], and several crops [12,42].

Meanwhile, GS has also become one of the innovations holding promise in genomics-assisted-breeding (GAB) [126,127], facilitating quick crop improvement without detailed study of individual loci [2,117]. GS enables crop breeders to explore and increase genetic gain per selection in a breeding program per unit breeding cycle, consequently enhancing speed and efficiency of breeding programs [72,128]. In GS, several cycles of selection are used to accumulate favorable alleles that are associated with desired phenotypes, although no causal association between a specific gene and a phenotype is established [35,129]. In GS, genome-wide HTP markers that are in LD with QTL are used to estimate their effects through optimum statistical models, before genomic estimated breeding values GEBVs are calculated for each individual to select potential elite lines [72,117,127,129]. In fact, two population types are needed in GS: a training population (also known as reference population) that is composed of a cohort of individuals with both genotypic and phenotypic data and a testing or (breeding) population that consists of candidate breeding lines with genotypic data only [127,129,130,131]. The predicted GEBVs are then used for selection, without the need for further phenotyping [72,114,132]. In this way, GS significantly shortens the breeding cycle as compared to conventional breeding methods. The utility of GS is that it can facilitate selection of complex traits including those for tolerance to drought, heat, cold, flooding, etc. [2]. Additionally, with GS, decisions on selections can be made during the off-season, resulting in genetic gain improvements on an annual basis [133]. Already, GS has shown to be an economical and viable alternative to MAS and phenotypic selection for quantitative traits and has fast-tracked crop breeding programs in cereal crops [72,127]. For these reasons, GS is suggested to hold great promise for adapting cereal crops to climate change [129].

## 5. Broadening Crop Genetic Diversity through Mutagenesis

The rigorous screening applied by crop breeders in the crop domestication process and eventual breeding of elite cultivars has resulted in considerable decline in natural genetic diversity. Consequently, this genetic erosion has become a bottleneck in further crop improvement efforts [134]. However, it is well known that the availability of heritable genetic variation is a prerequisite for any crop improvement program [30,135,136]. Therefore, broadening crop genetic base by induced mutations has become a common tool for creating genetic variation for use in crop improvement programs [108].

Aside from recombination, plant mutation induction by physical (via ionizing radiation such as X-rays, gamma rays, fast neutrons, etc.) and chemical mutagens (ethyl methane sulphonate, methyl methane sulphonate, sodium azide, etc.) is the most common approach for generating novel variations [136]. The resulting populations generated by physical and chemical mutagenesis are then screened for mutants with desirable phenotypes, and the genetic base underlying those phenotypes deciphered through mutant characterization [137]. Ion radiation induced mutations can increase the natural mutation rate by ~1 × 10^3^–1 × 10^6^-fold (www.iaea.org, accessed on 28 March 2021) and have been widely used to generate heritable genetic variability in the development of novel crop cultivars for the past century, generating billions of additional dollars in the process [108].

Whereas physical mutagens such as gamma rays and neutrons often result in large scale deletion of DNA and chromosomal structure alterations, chemical mutagens often affect single nucleotide pairs, with the extent of mutation being dependent on the tissue targeted, time and degree of exposure [136,138]. The aim in mutagenesis breeding is to cause maximal genetic variation with minimal decline in viability [134]. Therefore, mutations at single nucleotide pairs are particularly of much interest to crop breeders since large-scale alterations in chromosome structures usually result in serious deleterious consequences [138]. Application of mutation induction techniques has generated considerable amount of genetic variability, thereby playing an important role in plant breeding, genetics, and advanced genomics studies (www.iaea.org, accessed on 28 March 2021). Thus, mutagenesis-based crop improvement programs have benefited from new genetic variation and novel traits [108]. Consequently, the generated mutant crop cultivars contributed considerably to global food and nutrition security [139]. For instance, several mutant cultivars with enhanced productivity, abiotic stress tolerance, biotic stress resistance, and improved nutritive value have been developed in different cereal crops (Table 2, [136,140]).

Meanwhile, modern innovations in reverse genetics and gene discovery tools, coupled with recent advances in genome sequencing, bioinformatics and HTP technologies have brought heightened interest in the use of chemically induced mutations for crop improvement [145,146]. Particularly, the advent of TILLING (targeting induced local lesions in genomes) technology has revolutionized chemically-induced-mutagenesis-based crop breeding endeavors [134,147]. TILLING is a reverse genetics tool combining traditional chemical mutagenesis with high-throughput mismatch detection technique to identify series of single base pair allelic variations within a gene of interest [138,147]. This approach generates a wide range of mutant alleles, is fast and automatable, and is applicable to any organism that is amenable to chemical mutagenesis [148]. TILLING has been successfully applied in different cereal crop species including maize, wheat, rice, sorghum as comprehensively reviewed in previous articles [134,145,147].

Although TILLING populations have been conventionally used for reverse genetic approaches, current innovations in whole-genome sequencing have opened new opportunities for the identification of mutants in candidate genes, and the availability of sequence information from entire mutant population will permit the use of TILLING populations for forward genetic methodologies such as starting from an interesting phenotype detected in certain mutant lines to clone the underlying genes via association mapping [149]. For instance, the utility of TILLING technology has been proven in the cloning of wheat gene *Stb6* [150]. The gene *Stb6* encodes a conserved wall-associated receptor kinase (WAK) and confers pathogen resistance to the fungal *Septoria tritici* blotch (STB) disease. In a cultivar (Cadenza) harboring *Stb6* gene, Saintenac et al. [150] identified nonsense and mis-sense mutations for two of the candidate genes they had observed via genetic mapping. Mutations in a gene encoding WAK showed increased susceptibility compared to the wild-type control, confirming that this WAK gene is *Stb6* [150]. In the face of the changing global climate, TILLING technology holds new prospects for gene cloning disease resistance and abiotic stress genes in cereal crops [149]. For a detailed, and more recent, excellent review on TILLING in cereal crops for allele expansion and mutation detection, we refer you to Irshad et al. [151].

In cereal crops such as rice, insertional mutagenesis (IM) is an important tool for large-scale functional genomics analyses and gene discovery, using molecular tags such as T-DNA, *activator*/*dissociation* (*Ac/Ds*) insertions, transposons, or retrotransposons [152,153]. In instances where the identification of biological functions for redundant and vital genes is not feasible with the use of knockout mutants generated through chemical or physical mutagens [154], IM coupled with gene activation tagging technique plays a key role in circumventing such concern. The IM approach has been widely used to generate mutant libraries that allow for the easy identification of tagged genes by use of PCR-based techniques such as TAIL-PCR (thermal asymmetric interlaced PCR) or inverse PCR [152]. Generation of large mutant populations using IM approach is hindered by the approach’s stringent need for plant transformation methods and its low mutation frequency. However, in crops such as rice, innovations in the efficient plant transformation protocols and the availability of a wide range of transformation vectors have facilitated for the increased use of IM approach in rice functional genetic studies [152,155,156]. In particular, the IM approach has been successfully used in japonica cultivars, aided by the availability of reliable and reproducible *Agrobacterium tumefaciens*-mediated transformation techniques [155,157,158]. In this technique, the mutagen performs the role of a molecular tag, facilitating the identification of disrupted genes. When coupled with the desirable phenotype, the insertion tag will facilitate the isolation of the gene of interest [159]. For a detailed review on the use of IM approach in cereal crop functional genomics, we refer you to Ram et al. [152].

In relation to the future of plant mutagenesis, we hold the view that with the space explorations getting increasingly frequent, cosmic radiation induced mutations will become more important in generating novel allelic variations essential for strengthening crop breeders’ toolbox. It will be interesting to see how the new crop cultivars generated from such technologies will impact crop nutritional quality and human safety concerns.

## 6. Use of Sequence Specific Nucleases for Precise Gene Editing for Crop Improvements

Recent progress in genome engineering has revolutionized the precise editing of DNA sequences in living cells, thereby prompting targeted plant genetic manipulations for our benefit [160]. DNA can now be altered in various ways such as the introduction of specific nucleotide substitutions in a gene that alter a protein’s amino acid sequence, deletion of genes or chromosomal segments, and insertion of foreign DNA at specific genomic sites [161]. Such programmed DNA sequence modifications are facilitated by sequence-specific nucleases (SSNs) directed to modify target genes at desirable locations on the genome by creating double-strand breaks (DSBs) at specific genomic loci to be altered [162,163]. The double-stranded (ds) DNA is cleaved at particular loci by means of mainly three programmable SSNs, viz., zinc finger nucleases (ZFNs), transcriptional activator-like effector nucleases (TALENs), and CRISPR-Cas9 [161,164,165]. The DNA DSBs then undergo natural repair, via either homologous recombination (HR) or error-prone non-homologous end joining (NHEJ) [166,167,168]. These DSBs repair can be controlled to achieve the desired sequence modifications such as DNA deletions or insertions of larges arrays of transgenes [160]. For instance, the NHEJ repair mechanism causes mutation-like random insertions or deletions (InDels) or substitution [169]. If it occurs in the coding region of the gene, it may cause frame shift mutation, resulting in a target gene knockout [170]. On the other hand, the HR mechanism accurately repairs DNA DSBs by integrating a DNA donor containing homologous overhangs at the target site [166,171]. For detailed reviews on DNA DSBs repair mechanisms, we refer you to previous articles [164,167,172,173].

ZFNs are chimeric protein fusion of a non-specific DNA cleavage domain and a synthetic zinc finger-based domain that binds to DNA [164,171]. These ZFNs facilitate the creation of specific breaks in ds DNA, without pre-determined target site. When the target sequence specific array of zinc fingers is fused with endonuclease domain, usually a non-specific cleavage domain from Fok1, a type IIS restriction endonuclease, the breakage at a desired site can be formed [171]. Fok1 has an N-terminal DNA binding domain and a C-terminal domain possessing non-specific DNA cleavage activity. The DNA binding domain consists of zinc finger binding arrays (3–6 in number), with each binding array capable of recognizing a target DNA sequence of three consecutive nucleotides (3 base pairs) length [165]. In total, 9–12 nucleotides of the DNA sequence may be recognized per protein monomer [173]. Two Fok1 zinc finger binding arrays (each 18–24 bp in length and spaced by 5–8 bp) recognize respective sequences targeted (that are in close proximity on opposite strands) and dimerize (get aligned in reverse fashion with each other) [165]. This results in a staggered cut in the ds DNA; the spacing between the two zinc finger binding arrays is a critical design component of the ZFN, allowing for Fok1 monomers to dimerize and generate the much-needed DSB in the target DNA sequence. Consequently, a functional ZFN dimer has a recognition site of 18–24 nucleotides in length (excluding the spacer region) [165,173]. The high specificity of ZFNs has promoted their wide application in targeted gene editing in plants and animals [164,165].

Similar to ZFNs, a TALEN is a chimeric protein fusion of non-specific DNA cleavage domain of Fok1 nucleases with the DNA binding domain [164]. However, in TALENs, the DNA binding domain is a transcription activator-like effector (TALE) array [165,171,174]. In nature, TALENs are Type III effector proteins synthesized by *Xanthomonas* bacterial species to promote virulence in plants such as rice and cotton [175,176]. A TALE DNA binding domain is an array (of up to 30 copies) of highly conserved tandem repeats that span 34 amino acids each [173], with each repeat being able to recognize one nucleotide in the target sequence [165]. The tandem repeats on the TALE DNA binding domain are followed by a sequence of 20 amino acids, commonly known as half repeats [165]. Moreover, the TALE DNA binding domain possesses a simple repeat variable located only at two (12 and 13) positions known as repeat variable di-residue (RVD), whose function is to recognize a specific DNA sequence [165,170]. By targeting the sequence within the RVD (complementary to target sequence), the TALENs can be designed for a targeted site-specific DNA cleavage [170]. The mechanism of action of TALEN is the same as ZFNs, and the designing of TALEN is considerably easier as compared to ZFNs [165]. In terms of utility, TALENs boast greater specificity and efficiency and are considerably cheaper and quicker to assemble as compared to ZFNs [177,178]. Consequently, the TALEN approach has been used for precise genome editing and has shown great potential for various applications in biotechnology, synthetic biology, and crop improvement programs [164,170,173,178].

Among the three programmable SSNs based approaches used for genome editing, CRISPR-Cas9 represents a significant advance within the field of genetics and molecular biology [30,179] and has garnered much attention because of its great accuracy, quickness, adaptability, and simplicity [162,180,181]. The CRISPR-Cas9 system is a prokaryotic immunity system based on a targeted DNA-destroying defense system originally found in bacteria and archaea [164,177], aimed at defending organisms from invading viral and plasmid DNAs [166]. CRISPRs are small repeats (around 24–48 nucleotides in length) interspaced by foreign DNA from the earlier invaders against which protection is to be deployed. These spacers, which are known as protospacers, are always associated with a protospacer adjacent motif (PAM) [177]. Among the Cas effectors, class 2 effectors, such as Cas9 (type II) and Cpf1 (type V), are the most used in this defense system [182]. The principal components of the CRISPR/Cas9 system comprise the Cas9 protein, the trans-activating CRISPR RNA (tracrRNA) sequences, and the CRISPR RNA (crRNA) [166]. The tracrRNA and crRNA are engineered into single guide RNA (sgRNA) (of usually about 20 nucleotides [183], which is normally used in genome editing [177,184].

For its mechanism of action, the CRISPR-Cas9 system recruits an RNA-directed cleavage of foreign DNA to offer protection against the invading plasmids and viruses [177]. In simple terms, when bacteria detect the presence of viral DNA, they produce RNA corresponding to that of the invading virus. This RNA then recruits the Cas9 protein and guides it to the section of the genome complementary to the viral DNA, which the Cas9 protein will then cleave and eliminate from the bacterium’s genome [185]. Thus, co-expression of Cas9 and an engineered sgRNA forms a sequence-homology-dependent endonuclease that generates DNA DSBs within a specified target sequence along the genome [166,184]. The DNA DSBs can then be corrected by the cell’s endogenous DNA repair mechanism, either via NHEJ, possibly resulting in mutations, or through homology directed repair [179,183].

Since its discovery as ‘genetic scissors’ by Emmanuelle Charpentier of the Max Planck Unit (Berlin, Germany) and Jennifer A. Doudna of University of California (Berkeley, CA, USA) in 2012 [186], which eventually won them a Nobel Prize in Chemistry in November 2020 for ‘the development of a method for genome editing’ [185], CRISPR-Cas9 technology has evolved as the most powerful tool worldwide for precise genome editing and generation of genetic models for both fundamental and applied research [161,183,187,188]. Particularly, the ability of CRISPR-Cas9 system to localize a protein to a specific DNA sequence opened up several new opportunities, and the improvement of Cas9 has spawned several other potentially remarkable DNA manipulation tools and techniques [165,179], and it is now routinely employed to enable gene knockout, gene insertion, and gene replacement methods in different model genetic organisms [164,189,190,191]. Thus, CRISPR-Cas9 technology has been widely used to generate nutrition-improved and climate resilient cultivars in cereal crops (Table 3, [180,181,192,193,194,195]). For comparison amongst different SSNs, we refer you to previous detailed reviews [161,165,195].

However, despite the promise of the advanced technologies in gene editing and crop improvements for global food security and climate adaptation, public and scientific concerns related to ethics, and unsubstantiated human and environmental health and safety concerns brought about by genetically engineered crop cultivars (GECs) have been raised [164]. The resultant government regulatory frameworks aimed at safeguarding human and environmental health have imposed major cost barriers to the swift widespread adoption of newly developed GECs [140,206]. Going forward, the extent of these government regulations imposed on GECs will have a huge bearing on the cost of GECs’ further development itself and how rapidly they will be adopted for food and feed [68]. Additionally, the general public’s preparedness in embracing GEC-derived food products will also determine the extent of adoption of these gene editing approaches in crop improvement programs, especially in least developed countries where cereal crops are major providers of staple diets [207,208].

## 7. Double Haploid Technique as a Tool for Accelerated Crop Breeding for Climate Resilience

Speed breeding of climate-resilient and nutritionally superior crops targets to optimize and integrate the parameters that affect plant growth and reproduction to lessen generation times and the period taken to observe phenotypes, especially in the context of climate change [209]. Double haploid (DH) technology has made these speed breeding targets achievable. Double haploids (DHs) are plants derived from a single pollen grain and doubled artificially to form homozygous diploids [210], with a DH individual possessing two homologous chromosomes/genes [211], so that the amount of recombination information is equivalent to a backcross [210]. The utility of DH technology over conventional breeding approaches is that DH achieves complete homozygosity in a single generation, thereby significantly shortening the time required to produce pure lines [211]. Consequently, DH technology has had a significant impact on reducing time, labor and cost in crop improvement programs [212]. Additionally, because all individuals are homozygous, DHs can be transferred between different labs and environments for assessing the effect of the environment on gene expression [210]. Therefore, DHs are ideal for estimating QTL × environment interactions as complete homozygosity allows better estimates of trait means and facilitates for more accurate selection over locations and years [211].

The development of DH plants allows crop breeders to achieve homozygosity in segregating populations in a single generation as compared to 5–7 generations by conventional breeding methods. This permits selection of stable lines to start much earlier [210]. Therefore, DHs provide a time advantage for incorporation of quantitative traits that cannot be readily selected in the early segregating generations arising from conventional crosses [213], and they significantly reduce the size of populations needed to find a desired genotype [214]. Moreover, DHs provide an efficient screening material for desired mutants and other material for complex traits [214]. Therefore, combination of DH breeding approach with MAS and other new techniques such as directed in vitro mutagenesis and in vitro screening can be a vital tool for effective selection and efficient incorporation of complex traits such as drought, cold, and salinity tolerance [213,214]. Further, complete homozygosity facilitates more precise phenotyping and allows accurate gene-trait association in genetic mapping and gene function studies [211,215].

Despite the DH technology suffering from the limitations associated with anther/microspore culture (including low regeneration rate, high genotype-specific response, high frequency of callogenesis, and low recovery of DH plants) [214], there has been significant crop-specific protocols [140] and technology improvements that have facilitated its increased application in cultivar improvement, genetic mapping, mutagenesis, and gene functional analyses [211,216]. Thus, the DHs have been applied in genetic research and crop improvement efforts in cereal crops, including barley, maize, wheat, rice, and rye [211,213,217,218,219,220]. For instance, DHs created from haploid wheat plants developed by anther culture or fertilization with maize pollen have been used for wheat genetic research and breeding, whereby maize-pollen-derived haploids have been more feasible than anther culture in durum wheat; the rate of DH plant production in the durum × maize system has been significantly improved [213].

Another practical example is that DH technology has been successfully used in commercial maize breeding, to produce and double the chromosomes in maternal haploids to generate DHs (instant inbred lines), and paternal haploids produced through *indeterminate gametophyte1* mutation are utilized to convert male-fertile lines into cytoplasmic male-sterile lines [221,222]. Consequently, this has significantly reduced the breeding time from about 7–8 generations/seasons to two generations, thereby making the breeding of maize more efficient and economical [221]. Notably, since 2010, DHs have been progressively adopted in CIMMYT maize breeding programs, steadily substituting pedigree breeding. CIMMYT has since developed above 200,000 maize DH lines from diverse source populations and successfully recognized several maize DH lines harboring superior traits for use in maize breeding programs across Sub-Saharan African, Latin America, and Asia [223]. Going forward, the successful production of DHs on a routine basis would shorten cultivar development period and provide excellent recombinant inbred lines for molecular mapping applications [210].

## 8. The Integral Role of Crop Phenotyping in Complementing Crop Genotyping

Phenotyping encompasses measurement of observable traits that reflect the biological functioning of gene variants or alleles as influenced by the environment, and generally, phenotyping for crop improvement via breeding calls for assessment of hundreds or thousands of genetic lines [224]. In other words, phenotyping refers to the application of methodologies and protocols to quantify specific traits related to plant structure or function, with these traits ranging from cellular to whole-plant levels [225,226]. This means that such a trait that is subject to phenotyping can be any physiological, morphological, or phenological feature, from the cell to whole plant level [227]. Combining all these definitions, Yang et al. [228] have described crop phenomics as the multidisciplinary study of HTP accurate acquisition and analysis of multidimensional phenotypes on an organism-wide scale through crop development.

Phenotyping has become an integral component of the crop improvement programs by contributing towards dissecting the genetic bases of certain crop phenotypic traits, particularly those related to yield and stress tolerance [229,230,231]. For instance, in crop drought tolerance improvement programs, yield and yield attributing factors (the primary trait) are targeted for direct selection whilst secondary traits (those traits in addition to yield, such as root architecture, anthesis-silking-interval, stay green, leaf rolling, etc.) are vital in conferring drought tolerance and contributing to final yield indirectly [232,233,234]. Conventionally, phenotyping of secondary traits has involved field assessments of easily observable and scored morphological characters such as plant height, flowering date, leaf number, etc. However, researchers have since discovered that tolerance to abiotic stresses, such as drought, involves metabolic and regulatory functions, for which measurements of targeted processes will more likely offer valuable information regarding the underlying biology and have since developed better methods for assaying of such traits [224].

Several component traits of plants acclimation to environmental stresses are controlled quantitatively. Therefore, enhancing phenotyping accuracy has become more imperative to improve the heritability of these traits. Additionally, the target quantitative traits would require rapid and precise measurement [114]. Researchers have since used physiological phenotyping approaches in controlled-environment studies in the determination of the mechanistic basis of abiotic stress responses [224]. More importantly, the phenotypic data generated from physiological phenotyping has been utilized to identify QTLs or genes through QTL mapping, association mapping and GWAS for GAB for crop improvement [228]. However, despite their positive contributions, many of these physiological phenotyping approaches are commonly detailed, time-consuming, expensive, and can only be effectively applied to a limited number of genotypes [224,226,235]. Consequently, there has been a critical limitation in applying physiological information in crop improvement programs [236].

Precise physiological phenotyping of specific plant traits is crucial in enhancing crop breeding programs, although characterization of non-visual (physiological) traits is difficult and complicated [225,230]. Accurate phenotyping and effective integration of phenotyping in crop breeding programs will entail clearly specifying and differentiating the physiological phenotyping methodology at every breeding step. More specifically, there will be need to (i) find evidence that a hypothesized plant trait will lead to crop improvement, (ii) understand the underlying basic process of the trait to guide the development of physiological screens, and (iii) develop multi-tier phenotypic screens that provide insight about trait expression at various phases along the breeding process [225].

Meanwhile, in the last decade, the rapid advances in HTP crop genotyping techniques have not be met with corresponding pace with regards to crop phenotypic methods [225,229]. Without quality and effective data, the enormous genotyping data cannot be effectively used for crop improvement [237]. Promisingly, the recent advances in robotics, information technology, and data extraction and analysis, coupled with systems integration have revolutionized plant phenotyping [228], with HTP phenotyping platforms (HTPPs) being developed in plants to keep pace with significant advancement in genotyping techniques to enhance the efficiency of crop improvement programs [238]. Crop morphology and physiology can now be assessed non-destructively and repeatedly across whole plant populations and throughout development, speedily, and less costly [228]. Specifically, we can now record trait data via sophisticated non-invasive imaging, remote-sensing, spectroscopy, image analysis, robotics, high-performance computing facilities, and phenomics tools and databases. These tools include red-green-blue (RGB) imaging, magnetic resonance imaging (MRI) and positron emission tomography (PET), near-infrared (NIR) spectroscopy, canopy spectral reflectance (SR) and infrared thermography (IRT), nuclear magnetic resonance, hyperspectral imaging, laser imaging, 3D imaging, and geographical information systems (GIS), among others [228,230,239,240,241,242].

These new phenomics methodologies target to significantly reduce the time required to gather essential data on traits such as plant architecture, photosynthesis, growth, or biomass productivity on thousands of individual plants from weeks or days to hours [230,238]. For instance, the use of robotics for measuring large number of plants means that large numbers of genotypes could be readily phenotyped [243,244,245]. Such advances in crop phenotyping are anticipated to provide crop researchers with tools and knowledge essential for unlocking the information coded in plant genomes [238]. Therefore, HTP now provides an essential link in translating laboratory research to the field. This is vital in developing novel genotypes that incorporate gene(s) expressing promising trait(s) into breeding lines adapted to target field environments [246]. For example, 3D visual modelling can be used to determine the plasticity of the canopy architecture and to evaluate the architectural and physiological characteristics that contribute to the higher productivity of the super rice varieties under drought stress conditions [247].

Thus, image-based phenotyping is currently being deployed for crop growth and disease monitoring in main cereals using HTPPs [240,248,249]. Such HTPPs are capable of acquiring quantitative plant information from large populations by minimally invasive or non-invasive methods integrated into screening protocols. Moreover, HTPP methodologies are amenable to deployment for both field and controlled environments (greenhouse and growth chambers) phenotyping [225,250]. Though currently very expensive, upscaling the utilization of these HTPPs will eventually enhance our understanding of crop growth kinetics and aid us improve crop models for systems biology and breeding of climate resilient cereal crop cultivars.

## 9. Unlocking the Roles of Plant Long Non-Coding RNAs (lncRNAs) in Regulating Plant Stress Responses and Adaptation

Technical innovations in genomics and bioinformatics, particularly the extensive use of high-throughput sequencing technology, have facilitated the discovery of more transcriptional units lacking protein-coding potential [251,252]. Such RNA units are now known as non-protein coding RNAs (ncRNAs), and they include small RNAs (sRNAs, ranging between 18–30 nucleotides; nt), medium sized ncRNAs (31–200 nt), and long non-coding RNAs (lncRNAs, >200 nt) [253,254]. Particularly, micro RNAs (miRNAs) are usually 21 nt sRNAs which direct the degradation and inhibition of translation of the mRNA targets, thereby suppressing the target genes [254]. The growing interest in studying plant ncRNAs has led to the development of several databases and tools which harbor information for the identification and annotation of those plant ncRNAs [254,255].

Among the several ncRNAs, lncRNAs have attracted much attention in genomics and stress response studies [252,256,257]. The lncRNAs have been defined as a diverse class of RNA transcripts containing >200 nt, with little or no significant proteincoding ability, but possessing critical roles in diverse cellular processes and plant abiotic stress responses [252,253,256]. Generally, lncRNAs are transcribed by RNA polymerase II or III, as well as polymerase IV/V in plants [255,258], processed via splicing or non-splicing, polyadenylation or non-polyadenylation, and can be compartmentalized in the nucleus or cytoplasm [251].

Increasing body of evidence has shown that plant lncRNAs play vital roles ‘as biological regulators’ in diverse cellular processes, including cell differentiation, genomic imprinting, epigenetic modification, and stress response, among others [252,259,260,261,262,263]. Some plant lncRNAs act as primary transcripts of small regulatory RNAs such as miRNAs and siRNAs, as well as playing roles in phosphate homeostasis and protein re-localization [251]. Additionally, lncRNAs have recently been functionally characterized in plant stress response mechanisms [264,265,266,267]. For instance, using a deep transcriptomic sequencing approach, researchers identified 584 lncRNAs to be responsive to simulated drought stress in foxtail millet [268]. In another study, researchers used deep RNA sequencing approach to identify lncRNAs responsive to combined salinity and boron stress in a hyper-arid Lluteño maize landrace from the Atacama Desert. Consequently, 1710 lncRNAs were putatively responsive to the combined stresses [252]. Similarly, 98 drought-responsive lncRNAs were observed to regulate drought-responsive regulatory genes involved in various metabolic processes in rice [269], whereas 77 heat-responsive lncRNAs were identified to regulate cellular responses to heat stress in wheat [270]. Taken together, various lncRNAs play vital roles in modulating stress responses by acting as target mimics for different miRNAs that control the expression of stress-responsive target genes and transcriptional factors via up- and down- regulation or regulatory hubs for controlling several hormonal signaling pathways at transcriptional, post transcriptional, and epigenomic levels [253,256]. For extensive reviews on lncRNAs and their functional roles in plant biology and stress responses, we refer you to previous articles [253,256,262,271].

Since field crops are continuously exposed to a combination of different biotic and abiotic stresses, plants institute elaborate adaptive response mechanisms in response, via reprogramming their gene expression at the transcriptional, post-transcriptional, and post-translational levels [252,270]. Therefore, unlocking the exact roles played by lncRNAs in specific abiotic and biotic stresses will facilitate the designing of lncRNAs biomarkers relevant for engineering climate-resilient crop cultivars [256,272].

## 10. Pan-Genomics, Transposable Elements, and Machine Learning Hold Promise for Crop Improvement Getting into the Future

### 10.1. Pan-Genomics Facilitating Better Understanding and Utilization of Broader Crop Genetic Diversity for Accelerated Crop Improvement

Improving productivity and climate resilience of major cereal crops requires understanding the causal processes, exploring the extent, and exploiting the maximum possible abundance of genetic variation within the gene pools [273]. Generally, crop reference genome sequences have been the basis of crop genomic and genetic studies, providing insights into gene content, genomic variation, and the genetic foundation for most agronomic traits [2,118,274]. Traditionally, researchers have employed reference genomes to predominantly target SNPs for crop genomic diversity investigations. Precisely, the accessibility of reference genomes for the major crops has facilitated genome-wide analyses of SNPs and subsequent marker–trait linkage studies to connect genetic variation with phenotypic variation [273].

Meanwhile, genome structural variation (SV) has become increasingly acknowledged as a fundamental aspect of genomic diversity [2,275,276,277]. Recognizably, genetic variation, especially SV, can cause considerable variation of functional gene complement and gene content among individuals within the same species [273]. Main SVs comprise the copy number variants (CNVs) and presence absence variants (PAVs). CNVs refer to sequences that are present in a different number of copies between, whereas PAVs are sequences that exist in one genome and are absent in another [277,278].

Unfortunately, the reliance on resequencing approaches premised on a single reference genome has limited our capacity to detect genomic SVs and constrained our understanding of the genetic diversity in major crop species [273]. It is now widely accepted that a single crop reference genome is incapable of capturing the full landscape of genetic diversity of a species and hence cannot offer full insights of the crop’s diversity [279,280]. On the other hand, since pan-genomes usually contain within-species CNVs and PAVs, a pan-genome offers a complete genomic variation repertoire of a genus [280]. Therefore, pan-genome analysis is a more robust and comprehensive approach that provides a platform to capture gene content variation and evaluate the genetic diversity of a species via investigation of its entire genome repertoire, through sequencing of multiple individuals of the same species [273]. This presents unprecedented opportunities for crop improvement going forward.

A pan-genome has been defined as the sum total of genes of a biological clade, such as a species, comprising of a set of core genes that are shared by all individuals and a set of dispensable (or variable) genes that are partially shared or individual specific [280,281]. Initially coined for prokaryotes and popular in microbiological studies [281], pan-genome analysis is becoming increasingly common in plant genome studies as well [2,280]. Recently, crop pan-genomes have been published for maize [282], rice [283,284,285], wheat [286], and *Brassica* species [287,288], among others. For extensive reviews on crop pan-genomes, we refer you to recent excellent papers [273,279,280,289]. Therefore, a paradigm shift from single reference genome to pan-genome analysis approach for detecting genetic diversity within species will eliminate single-sample bias and allow for a better representation of crop genetic diversity [2,280,290].

SVs such as PAVs and CNVs play crucial roles in influencing important climate-relevant crop agronomic traits [273]. Particularly, the dispensable genome has been observed to harbor genes responsible for crop adaptation and survival under different biotic and abiotic environments [280], including head smut resistance in maize [291], phosphorus starvation in rice [292], and temperature extremes, among others [273]. Therefore, pan-genomic studies will facilitate dissection of the genetic basis of these major agronomical traits, thereby aiding linking of genetic variation with agronomic traits via QTL studies or GWAS, which is critical for crop improvement [273,279]. Moreover, understanding pan-genomics will facilitate accelerated exploitation of CWRs for increasing diversity within gene pools, thereby expanding the toolbox available for plant breeding and crop improvement efforts [52,289]. Collectively, pan-genomics, coupled with advanced genome sequencing techniques, will facilitate better understanding of the crop genetic diversity and identification of novel crop alleles [279], ultimately broadening genetic resources for accelerated crop improvement for stable higher yields and climate resilience.

### 10.2. Transposable Elements as Research Target for Decoding Crop Genomes and Understanding Crop Responses to Biotic and Abiotic Stresses

With the modern advances in genome sequencing technology and assembly algorithms, our capacity to decode the complexity and structure of genomes has been significantly improved [289]. Resultantly, previously unknown genome structures such as transposable elements (TEs) are becoming clearer. TEs, also known as ‘jumping genes’, are ubiquitous mobile DNA sequences that are found in both eukaryotic and prokaryotic genomes [293]; they comprise large portions of plant genomes (Table 1, [86]). Examples of TEs include long terminal repeats (LTRs), miniature inverted repeat TEs (MITEs), *Ac/Ds* elements, helitrons, *Enhancer*/*Suppressor of mutation* (*En/Spm)* elements, and *mutator* (*Mu*) elements [293,294,295]. Plant genomes, including crop species such as maize, are rich in TEs [296,297]. TE activity mediates large-scale chromosomal reorganizations [298,299], creates majority of insertions and deletions in crop genomes [300], and modifies the architecture and amount of gene product that is transcribed [273,301,302]. Notably, TE transposition may influence transcriptional activity of adjoining genes by controlling epigenomic profile of the region or by altering the relative location of regulatory elements [303]. It is not surprising that TEs are the major contributors of genome size variation among different species [295,304,305], and important causes of SVs [273,289].

The importance of TEs to crop phenotypes has been repeatedly shown. For instance, in maize, TEs were shown to provide/activate important allelic regulatory variation in gene response to several abiotic stresses [306]. Similarly, *Gypsy* retrotransposon-mediated aluminum tolerance was achieved in rice, through enhanced expression of the citrate transporter OsFRDL4 [307]. As pan-genomes become widely available for crop species, TEs will receive increasing attention in crop improvement programs [289]. More crucially, development of new tools for analyzing complex pan-genomes, encompassing comprehensive TE annotation, will facilitate our further understanding of TEs’ varied roles within crop genomes and connecting TE variation to phenotypes of agronomic importance [279,303].

### 10.3. Machine Learning as a Powerful Tool for Gene Function Prediction and High-Throughput Field and Stress Phenotyping

Crop genomics research is not simply about acquiring molecular phenotypes, but also leveraging powerful data mining and bioinformatics tools to predict and interpret these phenotypes [308]. Fortunately, machine (or deep) learning (ML) has been observed to be effective in accomplishing these tasks in recent years [308]. ML refers to a group of computerized modelling approaches that can study patterns from the data so that they make automatic decisions without programming explicit rules [309]. ML allows algorithms to interpret data by learning patterns through experience [310].

When using ML, problems in the field are categorized into either supervised or unsupervised [311,312]. Supervised learning aims to obtain a model which maps its predictors, such as DNA sequences, to target variables, such as histone marks [308]. Predicting regulatory and non-regulatory regions in the maize genome [313], plant stress phenotyping [314], and predicting diseases and nutritional deficiencies in soybean [315] are examples of supervised learning applications. On the other hand, if there is no specification about the outcome in the data set, then the problem becomes unsupervised learning [308], with clustering and feature extraction being examples [316].

Using ML to analyze enormous, varied, and formless datasets (such as those generated by photo imaging or sequencing) may offer considerable advantages over conventional analytical methods [310,317]. ML has been applied in many areas of genomics and phenomics research, including genome assembly and genome annotation [318]; large-scale data analysis to resolve complex biological problems in genomics, metabolomics, transcriptomics, proteomics, and systems biology [319,320]; the inference of gene regulatory networks [321,322]; identification of true SNPs in polyploid plant species [323]; and high-throughput field and crop stress phenotyping [309,318,324,325].

Crop scientists can use ML to model the flow of information from genomic DNA sequences to molecular phenotypes, and to identify functional variants in natural populations using ML models [308,312]. Additionally, the power of ML in synthetic biology can be unleashed to create novel genomic elements with desirable functions [308]. Particularly for crop breeders, ML will aid the identification of functional genomic regions of agronomic value by facilitating functional annotation of genomes and permitting real-time high-throughput phenotyping of agronomic traits in both controlled and open-field environments [310,312]. Moreover, ML can be integrated with genome sequencing and bioinformatics to predict transcriptional factor binding sites [326]. Previously, ML methodologies have been successfully employed to detect several features, including protein-coding genes, miRNAs, lncRNAs [327], polyadenylation sites [328], and *cis*-regulatory elements (CREs) [312,329].

Several crop databases that integrate the enormous volume of heterogeneous and unstructured genotypic and phenotypic data (Big Data) now provide valuable resource for crop breeders and opportunities to unravel novel trait-associated candidate genes [308]. However, retrieval, analysis and interpretation of such Big Data is challenging [309]. Fortunately, ML offers promising computational and analytical solutions for the integrative analysis of these enormous datasets on the Big-Data scale [131,312,319]. Additionally, using ML to infer the relationships between CREs and genes is a promising field for identifying previously unknown candidates for crop improvement stepping into the future [330]. Further, ML approaches will become more important for crop yield prediction, high-throughput crop stress phenotyping and climate change impact evaluation in agriculture [131,309,318,331].

**Figure 1:**
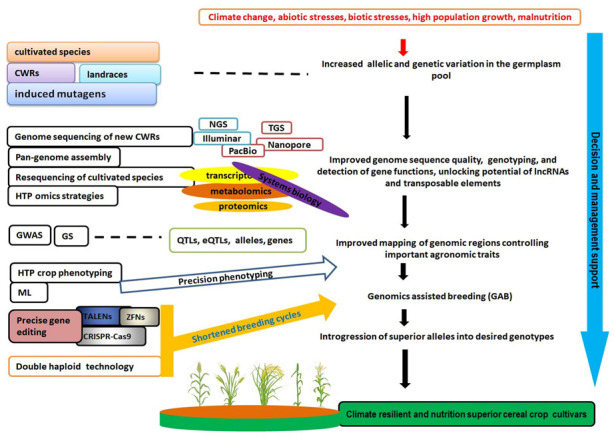
Hypothetical depiction of the role of plant genomics approaches in developing climate resilient and nutrition-superior cereal crop cultivars. An integration of genomics approaches with modern plant breeding, gene editing, crop phenotyping, and machine learning technologies ensures development of a comprehensive crop improvement program for climate resilient and nutrition-rich cereal crop cultivars. Abbreviations: CWRs, crop wild relatives; NGS, next generation sequencing; TGS, third generation sequencing; HTP, high throughput; GWAS, genome wide association studies; GS, genomic selection; ML, machine learning; ZFNs, zinc-finger nucleases; TALENs, transcriptional activator-like effector nucleases; QTL, quantitative trait loci; eQTL, expression quantitative trait loci.

Collectively, we have summarized the recent advances in crop genomics that are being applied to enhance cereal crop resilience to climate change as shown in Figure 1.

## 11. Conclusions

Modern developments in genome sequencing, assembly, and annotation, coupled with sophisticated bioinformatics and computational tools have facilitated our better understanding of the structure and information contained in crop genomes. Consequently, mapping of genomic regions controlling variation of target agronomic traits has been improved. Additionally, exploitation of an increased number of CWRs and orphaned species and the use of induced mutations are providing novel allelic diversity into the crop breeders’ toolbox. This has necessitated genomics-assisted breeding of climate-resilient crop cultivars. Further, the application of new gene editing techniques such as CRISPR-Cas9 and DH technology are accelerating the improvement of climate-resilient and nutrition-superior crop cultivars. Integration of ML and high-throughput crop phenotyping has become central in enhancing gene function prediction and linking genotypes to phenotypes. Going forward, unlocking the exact roles played by lncRNAs in specific abiotic and biotic stresses will facilitate the designing of lncRNA biomarkers relevant for engineering climate-resilient crop cultivars. Moreover, pan-genomics will enable our better decoding of the crop genetic diversity and identification of novel crop alleles, whereas comprehensive TE annotation and analysis will help us understand their varied roles within the crop genomes and link TE variation to phenotypes of agronomic importance. All these genomics strategies will be critical for breeding high-yielding, climate-resilient and highly nutritive cereal crops for the rising human population.

## Figures and Tables

**Table 2:** Selected examples of improved cereal crop cultivars generated by induced mutations (www.iaea.org (accessed on 28 March 2021), [136,141]).

Species Name	Mutant Name	Parent	Mutant Development Type (and Mutation Induction Type Used)	^1^ Trait Category	^2^ Description of Specific Traits Improved	^3^ Reg. Year	Country	References
*Oryza sativa* L.	Sinar 1	Sintanur	Gamma irradiation	Y, QNR	Higher yield and higher aromatic value than parent	2020	Indonesia	[142]
*Oryza sativa* L.	Sinar 2	Sintanur	Gamma irradiation	Y, BST, QNR	High yield, higher aromatic value, and higher disease resistance to BLB diseases	2020	Indonesia	[142]
*Oryza sativa* L.	Zhefu 802	Simei No. 2	Gamma irradiation	BST, Y, A, QNR	Higher rice blast resistance, higher yield, early maturity, good grain quality	1990	China	[139,141,142]
*Triticum aestivum* L.	Akebono-mochi	Kanto No. 107	Hybridization with mutant obtained by EMS chemical treatment	QNR	Amylose free, lower pasting temperature, higher peak viscosity, and higher breakdown than for non-waxy wheat	2000	Japan	[142,143]
*Triticum aestivum* L.	Binagom-1	L-880 (NIAB, Pakhistan)	Direct use of an induced mutant	AST, Y	Has higher salinity tolerance, higher yield	2016	Bangladesh	[142]
*Triticum aestivum* L.	Darkhan-172	Darkhan-95	Chemical mutageneis using sodium azide	Y, A	Higher yield, early maturity	2018	Mongolia	[142]
*Hordeum vulgare* L.	Centenario	Buenavista	Gamma irradiation (333 Gy)	A	Altered maturity, seed production traits	2006	Peru	[142]
*Hordeum vulgare* L.	Cruiser	Valticky, Diamant	Hybridization with mutant variety Diamant obtained by irradiation of seeds with X-rays (100 Gy)	A	Improved growth habit (erectoid type)	2001	Germany	[142]
*Hordeum vulgare* L.	Phenix	Kharkivskiy 99 (mutant)	Hybridization with mutant Kharkivskiy 99	AST	Improved drought tolerance	2000	Ukraine	[142]
*Zea mays* L.	Kneja 627	PCM4658	Hybridization with mutant (from the cross PCM4658 × Mo17)	Y, A	Improved grain (seed) yield, late maturity	2009	Bulgaria	[142]
*Zea mays* L.	P26	F1 P1 3747 SC M3	Treatment with fast neutrons (7.5 Gy)	A	Agronomic and botanic traits (combining ability)	2001	Hungary	[142]
*Zea mays* L.	Longfuyu 3	Fu2691 × 8008	Direct use of an induced mutant	BST	Improved resistance to bacterial diseases	2007	China	[142]
*Setaria* sp.	Jingu 21	Jinfen 52	Gamma irradiation (350 Gy)	Y, QNR	Improved grain (seed) yield, improved culinary quality	2000	China	[142]
*Panicum miliaceum* L.	Cheget	Mutant parents not specified.	Hybridization with two chemo mutants	AST, BST	Improved drought tolerance, improved smut resistance	1993	Russia	[142]
*Sorghum bicolor* L.	Fambe	CSM388	Direct use of an induced mutant, gamma irradiation (300 Gy)	AST, Y	Resistance to lodging, high grain yield (increased number of grains per panicle)	1998	Mali	[142]
*Sorghum bicolor* L.	PAHAT	-	Direct use of an induced mutant, gamma irradiation	Y, A, QNR	High yielding, semi dwarfness, early maturity, grain quality (protein, tannin, starch)	2013	Indonesia	[142,144]
*Sorghum bicolor* L.	Samurai 1	Zh-30	Direct use of an induced mutant, gamma irradiation (0.3 Other)	Y, A, BST, QNR, AST	High yield, improved food processing quality, improved biomass, lodging resistance, resistance to midrib rot disease, large seed size	2014	Indonesia	[142]

^1^ Trait category: AST, abiotic stress tolerance; BST, biotic stress tolerance; QNR, quality and nutrition-related; Y, yield; A, agronomic. ^2^ Description of trait improved: BLB diseases, bacterial late blight diseases. ^3^ Reg. year, year of registration with the Mutant Variety Database (www.mvd.iaea.org (accessed on 28 March 2021)), which may be later than the official release year within that variety’s country of development.

**Table 3:** Selected examples of significant gene targeting studies in cereal crops using CRISPR-Cas9 system [164,165,180,183].

Crop Species	Delivery Mode ^1^	Target Gene/s	DNA Repair Type ^2^	sgRNA Promoter	Cas9 Promoter ^3^	Vector Used	Trait Targeted for Improvement	References
*Oryza sativa* L.	EP	*ERF922*	NHEJ	OsU6a	Ubi	C-ERF922	Enhanced rice blast resistance	[196]
	AMT	*ALS*	HR	OsU3	Ubi	pCXUN-Cas9-gRNA1-gRNA2-armed donor vector	Improved herbicides resistance	[197]
	EP	*SBEIIb*	NHEJ	OsU3	Ubi	pCXUN-Cas9	High amylose content	[198]
	EP, AMT	*Gn1a*, *GS3*, *DEP1*	NHEJ	OsU6a	Ubi	pYLCRISPR/Cas9(I)	Improved grain number, larger grain size, and dense erect panicles	[199]
	AMT	*RR22*	NHEJ	OsU6a	Ubi	pYLCRSPR/Cas9 Pubi-H	Enhanced salt tolerance	[200]
	AMT	*PRX2*	NHEJ	OsPRX2	-	pCAMBIA1301	Improved potassium deficiency tolerance	[201]
*Triticum aestivum* L.	BMT	*GW2*	NHEJ	TaU6	Ubi	pET28a-Cas9-His	Increased grain weight and protein content	[202]
	BMT	*EDR1*	NHEJ	TaU6	Ubi	pJIT163-Ubi-Cas9	Increased powdery mildew resistance	[203]
	AMT	*MLO*	NHEJ	TaU6	Ubi	pUC-T vector (CWBIO)	Increased mildew resistance	[204]
	AMT	*DREB2 and ERF3*	NHEJ	TaU6	-	pJIT163-2NLSCas9	Improved drought resistance	[193]
*Zea mays* L.	AMT	*ARGOS8*	HR	ZmU6	Ubi	sgRNA-Cas9	Improved grain yield under drought stress tolerance	[194]
	AT	*ALS2*	HR	ZmU1	Ubi	UBI-Cas9 T-DNA vector	Improved resistance to herbicides	[205]
	BMT	*LIG1*, *M26*, *45, ALS1*	HR	ZmU6	Ubi	Cas9 DNA vector	Enhanced herbicide resistance and male sterility	[205]

^1^ Delivery Mode: EP, electroporation; AT, agrobacterium transformation; AMT, agrobacterium-mediated transformation; BT, biolistic transformation; BMT, biolistic mediated transformation. ^2^ DNA repair type: NHEJ, non-homologous end joining; HR, homologous recombination; ^3^ Ubi, ubiquitin promoter of Cas9.

## Data Availability

Not Applicable.

## References

[1] Bevan M., Waugh R. (2007). Applying plant genomics to crop improvement. Genome Biol..

[2] Scheben A., Yuan Y., Edwards D. (2016). Advances in genomics for adapting crops to climate change. Curr. Plant Biol..

[3] Hendre P.S., Muthemba S., Kariba R., Muchugi A., Fu Y., Chang Y., Song B., Liu H., Liu M., Liao X. (2019). African Orphan Crops Consortium (AOCC): Status of developing genomic resources for African orphan crops. Planta.

[4] Hunter D., Borelli T., Beltrame D.M., Oliveira C.N., Coradin L., Wasike V.W., Wasilwa L., Mwai J., Manjella A., Samarasinghe G.W. (2019). The potential of neglected and underutilized species for improving diets and nutrition. Planta.

[5] Sarwar M.H., Sarwar M.F., Sarwar M., Qadri N.A., Moghal S. (2013). The importance of cereals (Poacea: Gramineae) nutrition in human health: A review. J. Cereals Oilseeds.

[6] Fatima Z., Ahmed M., Hussain M., Abbas G., Ul-Allah S., Ahmad S., Ahmed N., Ali M.A., Sarwar G., ul Haque E. (2020). The fingerprints of climate warming on cereal crops phenology and adaptation options. Sci. Rep..

[7] Macauley H., Ramadjita T. (2015). Cereal Crops: Rice, Maize, Millet, Sorghum, Wheat: Background Paper, Feeding Africa, 21–23 October 2015, Dakar, Senegal.

[8] Shiferaw B., Prasanna B.M., Hellin J., Bänziger M. (2011). Crops that feed the world 6. Past successes and future challenges to the role played by maize in global food security. Food Secur..

[9] Godfray H.C.J., Beddington J.R., Crute I.R., Haddad L., Lawrence D., Muir J.F., Pretty J., Robinson S., Thomas S.M., Toulmin C. (2010). Food security: The challenge of feeding 9 billion people. Science.

[10] Gärtner P. Cereal Crops Fighting the Climate Chaos. (20 January 2021). https://phys.org/news/2021-01-cereal-crops-climate-chaos.html.

[11] Goron T.L., Raizada M.N. (2015). Genetic diversity and genomic resources available for the small millet crops to accelerate a New Green Revolution. Front. Plant Sci..

[12] Mohanta T.K., Bashir T., Hashem A., Abd_Allah E.F. (2017). Systems biology approach in plant abiotic stresses. Plant Physiol. Biochem..

[13] Ahmed K.F., Wang G., Yu M., Koo J., You L. (2015). Potential impact of climate change on cereal crop yield in West Africa. Clim. Chang..

[14] Wang J., Vanga S.K., Saxena R., Orsat V., Raghavan V. (2018). Effect of climate change on the yield of cereal crops: A review. Climate.

[15] Gustin G. Climate Change Could Lead to Major Crop Failures in World’s Biggest Corn Regions: Politics and Policy. Inside Climate News. (11 June 2018). https://insideclimatenews.org/news/11062018/climate-change-research-food-security-agriculture-impacts-corn-vegetables-crop-prices/.

[16] Reynolds M.P., Quilligan E., Aggarwal P.K., Bansal K.C., Cavalieri A.J., Chapman S.C., Chapotin S.M., Datta S.K., Duveiller E., Gill K.S. (2016). An integrated approach to maintaining cereal productivity under climate change. Glob. Food Secur..

[17] Ahsan F., Chandio A.A., Fang W. (2020). Climate change impacts on cereal crops production in Pakistan. Int. J. Clim. Chang. Strateg. Manag..

[18] Li M. (2018). Climate Change to Adversely Impact Grain Production in China by 2030.

[19] Eigenbrode S.D., Binns W.P., Huggins D.R. (2018). Confronting climate change challenges to dryland cereal production: A call for collaborative, transdisciplinary research, and producer engagement. Front. Ecol. Evol..

[20] Pourkheirandish M., Golicz A.A., Bhalla P.L., Singh M.B. (2020). Global role of crop genomics in the face of climate change. Front. Plant Sci..

[21] Qaim M. (2017). Globalisation of Agrifood Systems and Sustainable Nutrition. Proc. Nutr. Soc..

[22] Qaim M. (2020). Role of new plant breeding technologies for food security and sustainable agricultural development. Appl. Econ. Perspect. Policy.

[23] Kole C., Muthamilarasan M., Henry R., Edwards D., Sharma R., Abberton M., Batley J., Bentley A., Blakeney M., Bryant J. (2015). Application of genomics-assisted breeding for generation of climate resilient crops: Progress and prospects. Front. Plant Sci..

[24] Kahane R., Hodgkin T., Jaenicke H., Hoogendoorn C., Hermann M., Hughes J.D.A., Padulosi S., Looney N. (2013). Agrobiodiversity for food security, health and income. Agron. Sustain. Dev..

[25] Dawson I.K., Powell W., Hendre P., Bančič J., Hickey J.M., Kindt R., Hoad S., Hale I., Jamnadass R. (2019). The role of genetics in mainstreaming the production of new and orphan crops to diversify food systems and support human nutrition. New Phytol..

[26] Kilian B., Dempewolf H., Guarino L., Werner P., Coyne C., Warburton M.L. (2020). Crop Science special issue: Adapting agriculture to climate change: A walk on the wild side. Crop Sci..

[27] Ghatak A., Chaturvedi P., Bachmann G., Valledor L., Ramšak Ž., Bazargani M.M., Bajaj P., Jegadeesan S., Li W., Sun X. (2021). Physiological and Proteomic Signatures Reveal Mechanisms of Superior Drought Resilience in Pearl Millet Compared to Wheat. Front. Plant Sci..

[28] Ray D.K., Mueller N.D., West P.C., Foley J.A. (2013). Yield trends are insufficient to double global crop production by 2050. PLoS ONE.

[29] Biello D. (2011). Cereal killer: Climate Change Stunts Growth of Global Crop Yields. Sci. Am. Retrieved Jan..

[30] Gao C. (2021). Genome engineering for crop improvement and future agriculture. Cell.

[31] Mabhaudhi T., Chimonyo V.G.P., Hlahla S., Massawe F., Mayes S., Nhamo L., Modi A.T. (2019). Prospects of orphan crops in climate change. Planta.

[32] Cannarozzi G., Plaza-Wüthrich S., Esfeld K., Larti S., Wilson Y.S., Girma D., de Castro E., Chanyalew S., Blösch R., Farinelli L. (2014). Genome and transcriptome sequencing identifies breeding targets in the orphan crop tef (*Eragrostis tef*). BMC Genom..

[33] Chang Y., Liu H., Liu M., Liao X., Sahu S.K., Fu Y., Song B., Cheng S., Kariba R., Muthemba S. (2019). The draft genomes of five agriculturally important African orphan crops. GigaScience.

[34] Rodríguez J.P., Rahman H., Thushar S., Singh R.K. (2020). Healthy and resilient cereals and pseudo-cereals for marginal agriculture: Molecular advances for improving nutrient bioavailability. Front Genet..

[35] Bevan M.W., Uauy C. (2013). Genomics reveals new landscapes for crop improvement. Genome Biol..

[36] Campos-de Quiroz H. (2002). Plant genomics: An overview. Biol. Res..

[37] Terryn N., Rouzé P., Van Montagu M. (1999). Plant genomics. FEBS Lett..

[38] Akpınar B.A., Lucas S.J., Budak H. (2013). Genomics approaches for crop improvement against abiotic stress. Sci. World J..

[39] Singh R.K., Prasad A., Muthamilarasan M., Parida S.K., Prasad M. (2020). Breeding and biotechnological interventions for trait improvement: Status and prospects. Planta.

[40] Abdeeva I., Abdeev R., Bruskin S., Piruzian E. (2012). Transgenic plants as a tool for plant functional genomics. Transgenic Plants-Advances and Limitations.

[41] Singh B., Salaria N., Thakur K., Kukreja S., Gautam S., Goutam U. (2019). Functional Genomic Approaches to Improve Crop Plant Heat Stress Tolerance [version 1; peer review: 2 approved, 1 approved with reservations]. F1000Research.

[42] Bohra A., Chand Jha U., Godwin I.D., Kumar Varshney R. (2020). Genomic interventions for sustainable agriculture. Plant Biotechnol. J..

[43] Bansal K.C., Lenka S.K., Mondal T.K. (2014). Genomic resources for breeding crops with enhanced abiotic stress tolerance. Plant Breed..

[44] Kamenya S.N., Mikwa E.O., Song B., Odeny D.A. (2021). Genetics and breeding for climate change in Orphan crops. Theor. Appl. Genet..

[45] Gupta A., Rico-Medina A., Caño-Delgado A.I. (2020). The physiology of plant responses to drought. Science.

[46] Kumar A., Tomer V., Kaur A., Kumar V., Gupta K. (2018). Millets: A solution to agrarian and nutritional challenges. Agric. Food Secur..

[47] Ananda G.K.S., Myrans H., Norton S.L., Gleadow R., Furtado A., Henry R.J. (2020). Wild Sorghum as a Promising Resource for Crop Improvement. Front. Plant Sci..

[48] Choudhary M., Singh V., Muthusamy V., Wani S.H. (2017). Harnessing crop wild relatives for crop improvement. LS-An Int. J. Life Sci..

[49] Tuberosa R., Salvi S. (2006). Genomics-based approaches to improve drought tolerance of crops. Trends Plant Sci..

[50] Brozynska M., Furtado A., Henry R.J. (2016). Genomics of crop wild relatives: Expanding the gene pool for crop improvement. Plant Biotechnol. J..

[51] Kofsky J., Zhang H., Song B.-H. (2018). The Untapped Genetic Reservoir: The Past, Current, and Future Applications of the Wild Soybean (*Glycine soja*). Front. Plant Sci..

[52] Khan A.W., Garg V., Roorkiwal M., Golicz A.A., Edwards D., Varshney R.K. (2020). Super-pangenome by integrating the wild side of a species for accelerated crop improvement. Trends Plant Sci..

[53] Gupta S.C., de Wet M.J., Harlan J.R. (1978). Morphology of Saccharum- Sorghum hybrid derivatives. Am. J. Bot..

[54] Jannoo N., Grivet L., Chantret N., Garsmeur O., Glaszmann J.-C., Arruda P., D’Hont A. (2007). Orthologous comparison in a gene-rich region among grasses reveals stability in the sugarcane polyploid genome. Plant J..

[55] Dillon S.L., Shapter F.M., Henry R.J., Cordeiro G., Izquierdo L., Lee L.S. (2007). Domestication to crop improvement: Genetic resources for Sorghum and Saccharum (Andropogoneae). Ann. Bot..

[56] Mammadov J., Buyyarapu R., Guttikonda S.K., Parliament K., Abdurakhmonov I.Y., Kumpatla S.P. (2018). Wild Relatives of Maize, Rice, Cotton, and Soybean: Treasure Troves for Tolerance to Biotic and Abiotic Stresses. Front. Plant Sci..

[57] Warburton M.L., Rauf S., Marek L., Hussain M., Ogunola O., de Jesus Sanchez Gonzalez J. (2017). The use of crop wild relatives in maize and sunflower breeding. Crop Sci..

[58] Yumurtaci A. (2015). Utilization of wild relatives of wheat, barley, maize and oat in developing abiotic and biotic stress tolerant new varieties. Emir. J. Food Agric..

[59] Sharma D., Khulbe R.K., Pal R.S., Bettanaika J., Kant L. (2021). Wild Progenitor and Landraces Led Genetic Gain in the Modern-Day Maize (*Zea mays* L.). Landraces-Traditional Variety and Natural Breed.

[60] Abrouk M., Ahmed H.I., Cubry P., Šimoníková D., Cauet S., Pailles Y., Bettgenhaeuser J., Gapa L., Scarcelli N., Couderc M. (2020). Fonio millet Genome Unlocks African Orphan Crop Diversity for Agriculture in a Changing Climate. Nat. Commun..

[61] International Plant Genetic Resources Institute (IPGRI) (2002). Neglected and Underutilized Plant Species: Strategic Action Plan of the International Plant Genetic Resources Institute.

[62] Padulosi S., Hoeschle-Zeledon I. (2004). Underutilized plant species: What are they?. LEISA-LEUSDEN-.

[63] Chivenge P., Mabhaudhi T., Modi A.T., Mafongoya P. (2015). The potential role of neglected and underutilised crop species as future crops under water scarce conditions in Sub-Saharan Africa. Int. J. Environ. Res. Public Health.

[64] Varshney R.K., Ribaut J.M., Buckler E.S., Tuberosa R., Rafalski J.A., Langridge P. (2012). Can genomics boost productivity of orphan crops?. Nat. Biotechnol..

[65] Gregory P.J., Mayes S., Hui C.H., Jahanshiri E., Julkifle A., Kuppusamy G., Kuan H.W., Lin T.X., Massawe F., Suhairi T.A. (2019). Crops For the Future (CFF): An overview of research efforts in the adoption of underutilised species. Planta.

[66] Dansi A., Vodouhè R., Azokpota P., Yedomonhan H., Assogba P., Adjatin A., Loko Y.L., Dossou-Aminon I., Akpagana K. (2012). Diversity of the neglected and underutilized crop species of importance in Benin. Sci. World J..

[67] Mayes S., Ho W.K., Chai H.H., Gao X., Kundy A.C., Mateva K.I., Zahrulakmal M., Hahiree M.K.I.M., Kendabie P., Licea L.C. (2019). Bambara groundnut: An exemplar underutilised legume for resilience under climate change. Planta.

[68] Voytas D.F., Gao C. (2014). Precision genome engineering and agriculture: Opportunities and regulatory challenges. PLoS Biol..

[69] Lata C., Gupta S., Prasad M. (2013). Foxtail millet: A model crop for genetic and genomic studies in bioenergy grasses. Crit. Rev. Biotechnol..

[70] Rao P.P., Birthal P.S., Reddy B.V., Rai K.N., Ramesh S. (2006). Diagnostics of sorghum and pearl millet grains-based nutrition in India. Int. Sorghum Millets News Lett..

[71] Vadez V., Hash T., Bidinger F.R., Kholova J. (2012). Phenotyping pearl millet for adaptation to drought. Front. Physiol..

[72] Srivastava R.K., Singh R.B., Pujarula V.L., Bollam S., Pusuluri M., Chellapilla T.S., Yadav R.S., Gupta R. (2020). Genome-Wide Association Studies and Genomic Selection in Pearl Millet: Advances and Prospects. Front. Genet..

[73] Varshney R.K., Shi C., Thudi M., Mariac C., Wallace J., Qi P., Zhang H., Zhao Y., Wang X., Rathore A. (2017). *Pearl millet* genome sequence provides a resource to improve agronomic traits in arid environments. Nat. Biotech..

[74] Bennetzen J.L., Schmutz J., Wang H., Percifield R., Hawkins J., Pontaroli A.C., Estep M., Feng L., Vaughn J.N., Grimwood J. (2012). Reference genome sequence of the model plant Setaria. Nat. Biotechnol..

[75] Zhang G., Liu X., Quan Z., Cheng S., Xu X., Pan S., Xie M., Zeng P., Yue Z., Wang W. (2012). Genome sequence of foxtail millet (*Setaria italica*) provides insights into grass evolution and biofuel potential. Nat. Biotechnol..

[76] Batley J., Edwards D. (2016). The application of genomics and bioinformatics to accelerate crop improvement in a changing climate. Curr. Opin. Plant Biol..

[77] Varshney R.K., Hoisington D.A., Tyagi A.K. (2006). Advances in cereal genomics and applications in crop breeding. Trends Biotechnol..

[78] Li C., Lin F., An D., Wang W., Huang R. (2018). Genome Sequencing and Assembly by Long Reads in Plants. Genes.

[79] Sanger F., Coulson A.R. (1975). A rapid method for determining sequences in DNA by primed synthesis with DNA polymerase. J. Mol. Biol..

[80] Sanger F., Nicklen S., Coulson A.R. (1977). DNA sequencing with chain-terminating inhibitors. Proc. Natl. Acad. Sci. USA.

[81] Flagel L.E., Blackman B.K., Wendel J., Greilhuber J., Dolezel J., Leitch I. (2012). The First Ten Years of Plant Genome Sequencing and Prospects for the Next Decade. Plant Genome Diversity.

[82] Project, International Rice Genome Sequencing (2005). The map-based sequence of the rice genome. Nature.

[83] Schnable P.S., Ware D., Fulton R.S., Stein J.C., Wei F., Pasternak S., Liang C., Zhang J., Fulton L., Graves T.A. (2009). The B73 maize genome: Complexity, diversity, and dynamics. Science.

[84] Paterson A.H., Bowers J.E., Bruggmann R., Dubchak I., Grimwood J., Gundlach H., Haberer G., Hellsten U., Mitros T., Poliakov A. (2009). The sorghum bicolor genome and the diversifi cation of grasses. Nature.

[85] Metzker M.L. (2005). Emerging technologies in DNA sequencing. Genome Res..

[86] Michael T.P., Jackson S. (2013). The first 50 plant genomes. Plant Genome.

[87] Michael T.P., VanBuren R. (2015). Progress, challenges and the future of crop genomes. Curr. Opin. Plant Biol..

[88] McNally K.L., Mauleon R.P., Chebotarov D., Klassen S.P., Kohli A., Ye G., Leung H., Hamilton R.S., Wing R.A. (2020). Mass genome sequencing of crops and wild relatives to accelerate crop breeding: The digital rice genebank. IOP Conference Series, Proceedings of the Earth and Environmental Science, 1 March 2020, Bogor, Indonesia.

[89] Goodwin S., McPherson J.D., McCombie W.R. (2016). Coming of age: Ten years of next-generation sequencing technologies. Nat. Rev. Genet..

[90] McCormick R.F., Truong S.K., Sreedasyam A., Jenkins J., Shu S., Sims D., Kennedy M., Amirebrahimi M., Weers B.D., McKinley B. (2018). The *Sorghum bicolor* reference genome: Improved assembly, gene annotations, a transcriptome atlas, and signatures of genome organization. Plant J..

[91] Cooper E.A., Brenton Z.W., Flinn B.S., Jenkins J., Shu S., Flowers D., Luo F., Wang Y., Xia P., Barry K. (2019). A new reference genome for *Sorghum bicolor* reveals high levels of sequence similarity between sweet and grain genotypes: Implications for the genetics of sugar metabolism. BMC Genom..

[92] International Wheat Genome Sequencing Consortium (IWGSC) (2018). Wheat Genome: Shifting the limits in wheat research and breeding using a fully annotated reference genome. Science.

[93] Ling H.Q., Zhao S., Liu D., Wang J., Sun H., Zhang C., Fan H., Li D., Dong L., Tao Y. (2013). Draft genome of the wheat A-genome progenitor Triticum urartu. Nature.

[94] Li G., Wang L., Yang J., He H., Jin H., Li X., Ren T., Ren Z., Li F., Han X. (2021). A high-quality genome assembly highlights rye genomic characteristics and agronomically important genes. Nat. Genet..

[95] Hittalmani S., Mahesh H.B., Shirke M.D., Biradar H., Uday G., Aruna Y.R., Lohithaswa H.C., Mohanrao A. (2017). Genome and transcriptome sequence of finger millet (*Eleusine coracana* (L.) Gaertn.) provides insights into drought tolerance and nutraceutical properties. BMC Genom..

[96] International Barley Genome Sequencing Consortium (2012). A physical, genetic and functional sequence assembly of the barley genome. Nature.

[97] Wang X., Chen S., Ma X., Yssel A.E., Chaluvadi S.R., Johnson M.S., Gangashetty P., Hamidou F., Sanogo M.D., Zwaenepoel A. (2021). Genome sequence and genetic diversity analysis of an under-domesticated orphan crop, white fonio (*Digitaria exilis*). GigaScience.

[98] Rhoads A., Au K.F. (2015). PacBio sequencing and its applications. Genome Proteom. Bioinform..

[99] Berlin K., Koren S., Chin C.S. (2015). Assembling large genomes with single-molecule sequencing and locality-sensitive hashing. Nat. Biotechnol..

[100] MPerez-de-Castro A., Vilanova S., Cañizares J., Pascual L., MBlanca J., JDiez M., Prohens J., Picó B. (2012). Application of genomic tools in plant breeding. Curr. Genom..

[101] Cui J., Lu Z., Xu G., Wang Y., Jin B. (2020). Analysis and comprehensive comparison of PacBio and nanopore-based RNA sequencing of the Arabidopsis transcriptome. Plant Methods.

[102] Shi J., Ma X., Zhang J., Zhou Y., Liu M., Huang L., Sun S., Zhang X., Gao X., Zhan W. (2019). Chromosome confirmation capture resolved near complete genome assembly of broomcorn millet. Nat. Commun..

[103] Benevenuto J., Ferrão L.F.V., Amadeu R.R., Munoz P. (2019). How can a high-quality genome assembly help plant breeders?. Gigascience.

[104] Alqudah A.M., Sallam A., Baenziger P.S., Börner A. (2020). GWAS: Fast-forwarding gene identification and characterization in temperate Cereals: Lessons from Barley—A review. J. Adv. Res..

[105] Beyene Y., Semagn K., Crossa J., Mugo S., Atlin G.N., Tarekegne A., Meisel B., Sehabiague P., Vivek B.S., Oikeh S. (2016). Improving maize grain yield under drought stress and non-stress environments in sub-Saharan Africa using marker-assisted recurrent selection. Crop Sci..

[106] Ribeiro P.F., Badu-Apraku B., Gracen V.E., Danquah E.Y., Garcia-Oliveira A.L., Asante M.D., Afriyie-Debrah C., Gedil M. (2018). Identification of quantitative trait loci for grain yield and other traits in tropical maize under high and low soil-nitrogen environments. Crop Sci..

[107] Cattivelli L., Rizza F., Badeck F.W., Mazzucotelli E., Mastrangelo A.M., Francia E., Marè C., Tondelli A., Stanca A.M. (2008). Drought tolerance improvement in crop plants: An integrated view from breeding to genomics. Field Crops Res..

[108] Mousavi-Derazmahalleh M., Bayer P.E., Hane J.K., Valliyodan B., Nguyen H.T., Nelson M.N., Erskine W., Varshney R.K., Papa R., Edwards D. (2019). Adapting legume crops to climate change using genomic approaches. Plant Cell Environ..

[109] Choudhary M., Wani S.H., Kumar P., Bagaria P.K., Rakshit S., Roorkiwal M., Varshney R.K. (2019). QTLian breeding for climate resilience in cereals: Progress and prospects. Funct. Integr. Genom..

[110] Ahmad H.M., Azeem F., Tahir N., Iqbal M.S. (2018). QTL mapping for crop improvement against abiotic stresses in cereals. J. Anim. Plant Sci..

[111] Mir R.R., Zaman-Allah M., Sreenivasulu N., Trethowan R., Varshney R.K. (2012). Integrated genomics, physiology and breeding approaches for improving drought tolerance in crops. Theor. Appl. Genet..

[112] Liu S., Qin F. (2021). Genetic dissection of maize drought tolerance for trait improvement. Mol. Breed..

[113] Gupta S.M., Arora S., Mirza N., Pande A., Lata C., Puranik S., Kumar J., Kumar A. (2017). Finger Millet: A “Certain” Crop for an “Uncertain” Future and a Solution to Food Insecurity and Hidden Hunger under Stressful Environments. Front. Plant Sci..

[114] Nepolean T., Kaul J., Mukri G., Mittal S. (2018). Genomics-Enabled Next-Generation Breeding Approaches for Developing System-Specific Drought Tolerant Hybrids in Maize. Front. Plant Sci..

[115] Maazou A.R.S., Tu J.L., Qiu J., Liu Z.Z. (2016). Breeding for drought tolerance in maize (*Zea mays* L.). Am. J. Plant Sci..

[116] Pang Y., Liu C., Wang D., Amand P.S., Bernardo A., Li W., He F., Li L., Wang L., Yuan X. (2020). High-Resolution Genome-Wide Association Study Identifies Genomic Regions and Candidate Genes for Important Agronomic Traits in Wheat. Mol. Plant.

[117] Shamshad M., Sharma A. (2018). The usage of genomic selection strategy in plant breeding. Next Gener. Plant Breed..

[118] Huang X., Han B. (2014). Natural variations and genome-wide association studies in crop plants. Annu. Rev. Plant Biol..

[119] Rafalski J.A. (2010). Association genetics in crop improvement. Curr. Opin. Plant Biol..

[120] Jain M., Moharana K.C., Shankar R., Kumari R., Garg R. (2014). Genome wide discovery of DNA polymorphisms in rice cultivars with contrasting drought and salinity stress response and their functional relevance. Plant Biotechnol. J..

[121] Huang X., Wei X., Sang T., Zhao Q., Feng Q., Zhao Y., Li C., Zhu C., Lu T., Zhang Z. (2010). Genome-wide association studies of 14 agronomic traits in ricelandraces. Nat. Genet..

[122] Huang X., Zhao Y., Wei X., Li C., Wang A., Zhao Q., Li W., Guo Y., Deng L., Zhu C. (2012). Genome-wide association study of flowering time and grain yield traits in a worldwide collection of rice germplasm. Nat. Genet..

[123] Pham A.T., Maurer A., Pillen K., Brien C., Dowling K., Berger B., Eglinton J.K., March T.J. (2019). Genome-wide association of barley plant growth under drought stress using a nested association mapping population. BMC Plant Biol..

[124] Jia G., Huang X., Zhi H., Zhao Y., Zhao Q., Li W., Chai Y., Yang L., Liu K., Lu H. (2013). A haplotype map of genomic variations and genome-wide association studies of agronomic traits in foxtail millet (*Setaria italica*). Nat. Genet..

[125] Morris G.P., Ramu P., Deshpande S.P., Hash C.T., Shah T., Upadhyaya H.D., Riera- Lizarazu O., Brown P.J., Acharya C.B., Mitchell S.E. (2013). Population genomic and genome-wide association studies of agro climatic traits in sorghum. Proc. Natl. Acad. Sci..

[126] Liu X., Wang H., Hu X., Li K., Liu Z., Wu Y., Huang C. (2019). Improving Genomic Selection with Quantitative Trait Loci and Nonadditive Effects Revealed by Empirical Evidence in Maize. Front. Plant Sci..

[127] Voss-Fels K.P., Cooper M., Hayes B.J. (2019). Accelerating crop genetic gains with genomic selection. Theor. Appl. Genet..

[128] Spindel J., Begum H., Akdemir D., Virk P., Collard B., Redoña E., Atlin G., Jannink J.-L., McCouch S.R. (2015). Genomic selection and association mapping in rice (*Oryza sativa*): Effect of trait genetic architecture, training population composition, marker number and statistical model on accuracy of rice genomic selection in elite, tropical rice breeding lines. PLoS Genet..

[129] Xu Y., Liu X., Fu J., Wang H., Wang J., Huang C., Prasanna B.M., Olsen M.S., Wang G., Zhang A. (2020). Enhancing genetic gain through genomic selection: From livestock to plants. Plant Commun..

[130] Wang X., Xu Y., Hu Z., Xu C. (2018). Genomic selection methods for crop improvement: Current status and prospects. Crop J..

[131] Tong H., Nikoloski Z. (2021). Machine learning approaches for crop improvement: Leveraging phenotypic and genotypic big data. J. Plant Physiol..

[132] Meuwissen T.H., Hayes B.J., Goddard M.E. (2001). Prediction of total genetic value using genome-wide dense marker maps. Genetics.

[133] Heffner E.L., Sorrells M.E., Jannink J.L. (2009). Genomic selection for crop improvement. Crop Sci..

[134] Sikora P., Chawade A., Larsson M., Olsson J., Olsson O. (2011). Mutagenesis as a tool in plant genetics, functional genomics, and breeding. Int. J. Plant Genom..

[135] Muñoz-Amatriaín M., Cuesta-Marcos A., Hayes P.M., Muehlbauer G.J. (2014). Barley genetic variation: Implications for crop improvement. Brief. Funct. Genom..

[136] Jankowicz-Cieslak J., Mba C., Till B.J. (2017). Mutagenesis for crop breeding and functional genomics. Biotechnologies for Plant Mutation Breeding.

[137] Li J., Yang J., Li Y., Ma L. (2020). Current strategies and advances in wheat biology. Crop J..

[138] Singh R., Tiwari R., Sharma D., Tiwari V., Sharma I. (2014). Mutagenesis for wheat improvement in the genomics era. J. Wheat Res. (JWR).

[139] Kharkwal M.C., Shu Q.Y. (2009). The role of induced mutations in world food security. Induced plant mutations in the genomics era. Food Agric. Organ. United Nations Rome.

[140] Ahmar S., Gill R.A., Jung K.-H., Faheem A., Qasim M.U., Mubeen M., Zhou W. (2020). Conventional and Molecular Techniques from Simple Breeding to Speed Breeding in Crop Plants: Recent Advances and Future Outlook. Int. J. Mol. Sci..

[141] Pathirana R. (2011). Plant mutation breeding in agriculture. Plant Sci. Rev..

[142] Joint FAO/International Atomic Energy Agency (IAEA) Programme of Nuclear Techniques in Agriculture (2016). Mutant Variety Database (MVD). https://www.mvd.iaea.org/.

[143] Yamaguchi I., Otobe C.K., Yanagisawa T. (2003). Breeding of 2 waxy wheat [*Triticum aestivum*] cultivars, Akebono-mochi and Ibuki-mochi, and their main features. Bull. Natl. Inst. Crop Sci..

[144] Wanga M.A., Kumar A.A., Kangueehi G.N., Shimelis H., Horn L.N., Sarsu F., Andowa J.F. (2018). Breeding sorghum using induced mutations: Future prospect for Namibia. Am. J. Plant Sci..

[145] Kurowska M., Daszkowska-Golec A., Gruszka D., Marzec M., Szurman M., Szarejko I., Maluszynski M. (2011). TILLING-a shortcut in functional genomics. J. Appl. Genet..

[146] Fruzangohar M., Kalashyan E., Kalambettu P., Ens J., Wiebe K., Pozniak C.J., Tricker P.J., Baumann U. (2019). Novel Informatic Tools to Support Functional Annotation of the durum wheat genome. Front. Plant Sci..

[147] Chen L., Hao L., Parry M.A., Phillips A.L., Hu Y.G. (2014). Progress in TILLING as a tool for functional genomics and improvement of crops. J. Integr. Plant Biol..

[148] McCallum C., Henikoff S., Colbert T. (2004). Fred Hutchinson Cancer Research Center, Assignee. Reverse Genetic Strategy for Identifying Functional Mutations in Genes of Known Sequences. U.S. Patent.

[149] Bettgenhaeuser J., Krattinger S.G. (2019). Rapid gene cloning in cereals. Theor. Appl. Genet..

[150] Saintenac C., Lee W.S., Cambon F., Rudd J.J., King R.C., Marande W., Powers S.J., Bergès H., Phillips A.L., Uauy C. (2018). Wheat receptor-kinase-like protein Stb6 controls gene-for-gene resistance to fungal pathogen *Zymoseptoria tritici*. Nat. Genet..

[151] Irshad A., Guo H., Zhang S., Liu L. (2020). TILLING in cereal crops for allele expansion and mutation detection by using modern sequencing technologies. Agronomy.

[152] Ram H., Soni P., Salvi P., Gandass N., Sharma A., Kaur A., Sharma T.R. (2019). Insertional mutagenesis approaches and their use in rice for functional genomics. Plants.

[153] Kim S.Y., Kim C.K., Kang M., Ji S.U., Yoon U.H., Kim Y.H., Lee G.S. (2018). A Gene Functional Study of Rice Using Ac/Ds Insertional Mutant Population. Plant Breed. Biotech..

[154] Springer P.S. (2000). Gene traps: Tools for plant development and genomics. Plant Cell.

[155] Hiei Y., Ohta S., Komari T., Kumashiro T. (1994). Efficient transformation of rice (*Oryza sativa* L.) mediated by Agrobacterium and sequence analysis of the boundaries of the T-DNA. Plant J..

[156] Ratanasut K., Rod-In W., Sujipuli K. (2017). In planta Agrobacterium-mediated transformation of rice. Rice Sci..

[157] Cheng X., Sardana R.K., Altosaar I., Cunningham C., Porter A.J.R. (1998). Rice Transformation by Agrobacterium Infection. Recombinant Proteins from Plants. Methods in Biotechnology.

[158] Wu J.-L., Wu C., Lei C., Baraoidan M., Bordeos A., Madamba M.R.S., Ramos-Pamplona M., Mauleon R., Portugal A., Ulat V.J. (2005). Chemical- and irradiation-induced mutants of indica rice IR64 for forward and reverse genetics. Plant Mol. Biol..

[159] Hwang H.H., Yu M., Lai E.M. (2017). Agrobacterium-Mediated Plant Transformation: Biology and Applications. Arab. Book.

[160] Voytas D.F. (2013). Plant genome engineering with sequence-specific nucleases. Annu. Rev. Plant Biol..

[161] Sun Y., Li J., Xia L. (2016). Precise genome modification via sequence-specific nucleases-mediated gene targeting for crop improvement. Front. Genet..

[162] Weeks D.P., Spalding M.H., Yang B. (2016). Use of designer nucleases for targeted gene and genome editing in plants. Plant Biotechnol. J..

[163] Hilscher J., Bürstmayr H., Stoger E. (2017). Targeted modification of plant genomes for precision crop breeding. Biotechnol. J..

[164] Zhang Y., Massel K., Godwin I.D., Gao C. (2018). Applications and potential of genome editing in crop improvement. Genome Biol..

[165] Ansari W.A., Chandanshive S.U., Bhatt V., Nadaf A.B., Vats S., Katara J.L., Sonah H., Deshmukh R. (2020). Genome editing in cereals: Approaches, applications and challenges. Int. J. Mol. Sci..

[166] Jun R.E.N., Xixun H.U., Kejian W.A.N.G., Chun W.A.N.G. (2019). Development and application of CRISPR/Cas system in rice. Rice Sci..

[167] Wright W.D., Shah S.S., Heyer W.D. (2018). Homologous recombination and the repair of DNA double-strand breaks. J. Biol. Chem..

[168] Miglani G.S. (2017). Genome editing in crop improvement: Present scenario and future prospects. J. Crop Improv..

[169] Mladenov E., Iliakis G. (2011). Induction and repair of DNA double strand breaks: The increasing spectrum of non-homologous end joining pathways. Mutat. Res..

[170] Bhutia K.L., Tyagi W. (2017). Use of Sequence Specific Nucleases for Site Specific Modification of Plant Genome for Crop Improvement. Int. J. Agric. Sci. Res. (IJASR).

[171] Cristea S., Freyvert Y., Santiago Y., Holmes M.C., Urnov F.D., Gregory P.D., Cost G.J. (2013). In vivo cleavage of transgene donors promotes nuclease-mediated targeted integration. Biotechnol. Bioeng..

[172] Verma P., Tandon R., Yadav G., Gaur V. (2020). Structural aspects of DNA repair and recombination in crop improvement. Front. Genet..

[173] Puchta H., Fauser F. (2014). Synthetic nucleases for genome engineering in plants: Prospects for a bright future. Plant J..

[174] Joung J.K., Sander J.D. (2013). TALENs: A widely applicable technology for targeted genome editing. Nat. Rev. Mol. Cell. Biol..

[175] Ryan R.P., Vorhölter F.J., Potnis N., Jones J.B., Van Sluys M.A., Bogdanove A.J., Dow J.M. (2011). Pathogenomics of Xanthomonas: Understanding bacterium-plant interactions. Nat. Rev. Microbiol..

[176] Üstün S., Börnke F. (2014). Interactions of Xanthomonas type-III effector proteins with the plant ubiquitin and ubiquitin-like pathways. Front. Plant Sci..

[177] Ahmad N., Mukhtar Z. (2017). Genetic manipulations in crops: Challenges and opportunities. Genomics.

[178] Khan Z., Khan S.H., Mubarik M.S., Sadia B., Ahmad A. (2017). Use of TALEs and TALEN technology for genetic improvement of plants. Plant Mol. Biol. Rep..

[179] Ng W.A., Ma A., Chen M., Reed B.H. (2020). A method for rapid selection of randomly induced mutations in a gene of interest using CRISPR/Cas9 mediated activation of gene expression. G3 Genes Genomes Genet..

[180] Razzaq A., Saleem F., Kanwal M., Mustafa G., Yousaf S., Imran Arshad H.M., Hameed M.K., Khan M.S., Joyia F.A. (2019). Modern trends in plant genome editing: An inclusive review of the CRISPR/Cas9 toolbox. Int. J. Mol. Sci..

[181] Raza A., Tabassum J., Kudapa H., Varshney R.K. (2021). Can omics deliver temperature resilient ready-to-grow crops?. Crit. Rev. Biotechnol..

[182] Zetsche B., Gootenberg J.S., Abudayyeh O.O., Slaymaker I.M., Makarova K.S., Essletzbichler P., Volz S.E., Joung J., Van Der Oost J., Regev A. (2015). Cpf1 is a single RNA-guided endonuclease of a class 2 CRISPR-Cas system. Cell.

[183] Song G., Jia M., Chen K., Kong X., Khattak B., Xie C., Li A., Mao L. (2016). CRISPR/Cas9: A powerful tool for crop genome editing. Crop J..

[184] Jinek M., Chylinski K., Fonfara I., Hauer M., Doudna J.A., Charpentier E. (2012). A programmable dual-RNA–guided DNA endonuclease in adaptive bacterial immunity. Science.

[185] Valavanidis A. (2020). Nobel Prize in Chemistry 2020. Discovery of CRISPR-Cas9 Genetic Scissors. A revolutionary genome editing technology that can cut any DNA molecule at a predetermined site (10 November 2020). Sci. Rev..

[186] Boglioli E., Richard M. (2015). Rewriting the book of life: A new era in precision gene editing. Working Paper. Boston Consult. Group (BCG).

[187] Nadakuduti S.S., Enciso-Rodríguez F. (2021). Advances in Genome Editing With CRISPR Systems and Transformation Technologies for Plant DNA Manipulation. Front. Plant Sci..

[188] Mao Y., Zhang H., Xu N., Zhang B., Gou F., Zhu J.K. (2013). Application of the CRISPR–Cas system for efficient genome engineering in plants. Mol. Plant..

[189] Schaeffer S.M., Nakata P.A. (2015). CRISPR/Cas9-mediated genome editing and gene replacement in plants: Transitioning from lab to field. Plant Sci..

[190] Joung J., Konermann S., Gootenberg J.S., Abudayyeh O.O., Platt R.J., Brigham M.D., Sanjana N.E., Zhang F. (2017). Genome-scale CRISPR-Cas9 knockout and transcriptional activation screening. Nat. Protoc..

[191] Campenhout C.V., Cabochette P., Veillard A.C., Laczik M., Zelisko-Schmidt A., Sabatel C., Dhainaut M., Vanhollebeke B., Gueydan C., Kruys V. (2019). Guidelines for optimized gene knockout using CRISPR/Cas9. BioTechniques.

[192] Xu R., Yang Y., Qin R., Li H., Qiu C., Li L. (2016). Rapid improvement of grain weight via highly efficient CRISPR/Cas9-mediated multiplex genome editing in rice. J. Genet. Genom..

[193] Kim D., Kim D., Alptekin B., Budak H. (2017). CRISPR/Cas9 genome editing in wheat. Funct. Integr. Genom..

[194] Shi J., Gao H., Wang H., Lafitte H.R., Archibald R.L., Yang M., Hakimi S.M., Mo H., Habben J.E. (2017). ARGOS8 variants generated by CRISPR-Cas9 improve maize grain yield under field drought stress conditions. Plant Biotechnol. J..

[195] Kaul T., Sony S.K., Verma R., Motelb K.F.A., Prakash A.T., Eswaran M., Bharti J., Nehra M., Kaul R. (2020). Revisiting CRISPR/Cas-mediated crop improvement: Special focus on nutrition. J. Biosci..

[196] Wang F., Wang C., Liu P., Lei C., Hao W., Gao Y., Liu Y.G., Zhao K. (2016). Enhanced rice blast resistance by CRISPR/Cas9-targeted mutagenesis of the ERF transcription factor gene OsERF922. PLoS ONE.

[197] Butt H., Eid A., Ali Z., Atia M.A., Mokhtar M.M., Hassan N., Lee C.M., Bao G., Mahfouz M.M. (2017). Efficient CRISPR/Cas9-mediated genome editing using a chimeric single-guide RNA molecule. Front. Plant Sci..

[198] Sun Y., Jiao G., Liu Z., Zhang X., Li J., Guo X., Du W., Du J., Francis F., Zhao Y. (2017). Generation of high-amylose rice through CRISPR/Cas9-mediated targeted mutagenesis of starch branching enzymes. Front. Plant Sci..

[199] Li M., Li X., Zhou Z., Wu P., Fang M., Pan X., Lin Q., Luo W., Wu G., Li H. (2016). Reassessment of the four yield-related genes Gn1a, DEP1, GS3, and IPA1 in rice using a CRISPR/Cas9 system. Front. Plant Sci..

[200] Zhang A., Liu Y., Wang F., Li T., Chen Z., Kong D., Bi J., Zhang F., Luo X., Wang J. (2019). Enhanced rice salinity tolerance via CRISPR/Cas9-targeted mutagenesis of the OsRR22 gene. Mol. Breed..

[201] Mao X., Zheng Y., Xiao K., Wei Y., Zhu Y., Cai Q., Chen L., Xie H., Zhang J. (2018). OsPRX2 contributes to stomatal closure and improves potassium deficiency tolerance in rice. Biochem. Biophys. Res. Commun..

[202] Liang Z., Chen K., Li T., Zhang Y., Wang Y., Zhao Q., Liu J., Zhang H., Liu C., Ran Y. (2017). Efficient DNA-free genome editing of bread wheat using CRISPR/Cas9 ribonucleoprotein complexes. Nat. Commun..

[203] Zhang Y., Bai Y., Wu G., Zou S., Chen Y., Gao C., Tang D. (2017). Simultaneous modification of three homoeologs of TaEDR1 by genome editing enhances powdery mildew resistance in wheat. Plant J..

[204] Shan Q., Wang Y., Li J., Gao C. (2014). Genome editing in rice and wheat using the CRISPR/Cas system. Nat. Protoc..

[205] Svitashev S., Young J.K., Schwartz C., Gao H., Falco S.C., Cigan A.M. (2015). Targeted mutagenesis, precise gene editing, and site-specific gene insertion in maize using Cas9 and guide RNA. Plant Physiol..

[206] Prado J.R., Segers G., Voelker T., Carson D., Dobert R., Phillips J., Cook K., Cornejo C., Monken J., Grapes L. (2014). Genetically engineered crops: From idea to product. Annu. Rev. Plant Biol..

[207] Edmeades G.O. (2013). Progress in Achieving and Delivering Drought Tolerance in Maize—An Update.

[208] Zenda T., Liu S., Duan H. (2020). Adapting Cereal Grain Crops to Drought Stress: 2020 and Beyond. Abiotic Stress in Plants.

[209] Hickey L.T., Hafeez A.N., Robinson H., Jackson S.A., Leal-Bertioli S.C., Tester M., Gao C., Godwin I.D., Hayes B.J., Wulff B.B. (2019). Breeding crops to feed 10 billion. Nat. Biotechnol..

[210] Khan T.N., Meldrum A., Croser J.S., Wrigley C., Corke H., Seetharaman K., Faubion J. (2016). Pea: Overview. Encyclopedia of Food Grains.

[211] Yan G., Liu H., Wang H., Lu Z., Wang Y., Mullan D., Hamblin J., Liu C. (2017). Accelerated generation of selfed pure line plants for gene identification and crop breeding. Front. Plant Sci..

[212] Rajcan I., Boersma J.G., Shaw E.J., Moo-Young M. (2011). Plant Systems/Plant Genetic Techniques: Plant Breeder’s Toolbox. Comprehensive Biotechnology.

[213] Royo C., Elias E.M., Manthey F.A., Carena M. (2009). Durum Wheat Breeding. Cereals. Handbook of Plant Breeding.

[214] Gupta S.K. (2016). Brassicas. Breeding Oilseed Crops for Sustainable Production.

[215] Yang J., Liu Z., Chen Q., Qu Y., Tang J., Lübberstedt T., Li H. (2020). Mapping of QtL for Grain Yield components Based on a DH population in Maize. Sci. Rep..

[216] Hussain B., Kha M., Ali Q., Shaukat S. (2012). Double haploid production is the best method for genetic improvement and genetic studies of wheat. Int. J. Agro Vet. Med. Sci..

[217] Dwivedi S.L., Britt A.B., Tripathi L., Sharma S., Upadhyaya H.D., Ortiz R. (2015). Haploids: Constraints and opportunities in plant breeding. Biotechnol. Adv..

[218] Li H., Singh R.P., Braun H., Pfeiffer W.H., Wang J. (2013). Doubled haploids versus conventional breeding in CIMMYT wheat breeding programs. Crop Sci..

[219] Asif M. (2013). Progress and Opportunities of Doubled Haploid Production.

[220] Forster B.P., Thomas W.T.B. (2005). Doubled haploids in genetics and plant breeding. Plant Breed Rev..

[221] Weber D.F. (2014). Today’s use of haploids in corn plant breeding. Adv. Agron..

[222] Uliana Trentin H., Frei U.K., Lübberstedt T. (2020). Breeding maize maternal haploid inducers. Plants.

[223] Prasanna B.M., Cairns J.E., Zaidi P.H., Beyene Y., Makumbi D., Gowda M., Magorokosho C., Zaman-Allah M., Olsen M., Das A. (2021). Beat the stress: Breeding for climate resilience in maize for the tropical rainfed environments. Theor. Appl. Genet..

[224] Setter T.L. (2012). Analysis of constituents for phenotyping drought tolerance in crop improvement. Front. Physiol..

[225] Ghanem M.E., Marrou H., Sinclair T.R. (2015). Physiological phenotyping of plants for crop improvement. Trends Plant Sci..

[226] Fiorani F., and Schurr U. (2013). Future scenarios for plant phenotyping. Annu. Rev. Plant Biol..

[227] Violle C., Navas M.L., Vile D., Kazakou E., Fortunel C., Hummel I., Garnier E. (2007). Let the concept of trait be functional!. Oikos.

[228] Yang W., Feng H., Zhang X., Zhang J., Doonan J.H., Batchelor W.D., Xiong L., Yan J. (2020). Crop phenomics and high-throughput phenotyping: Past decades, current challenges, and future perspectives. Mol. Plant.

[229] Araus J.L., Cairns J.E. (2014). Field high-throughput phenotyping: The new crop breeding frontier. Trends Plant Sci..

[230] Großkinsky D.K., Svensgaard J., Christensen S., Roitsch T. (2015). Plant phenomics and the need for physiological phenotyping across scales to narrow the genotype-to-phenotype knowledge gap. J. Exp. Bot..

[231] Oladosu Y., Rafii M.Y., Samuel C., Fatai A., Magaji U., Kareem I., Kamarudin Z.S., Muhammad I., Kolapo K. (2019). Drought resistance in rice from conventional to molecular breeding: A Review. Int. J. Mol. Sci..

[232] Calleja-Cabrera J., Boter M., Oñate-Sánchez L., Pernas M. (2020). Root growth adaptation to climate change in crops. Front. Plant Sci..

[233] Khadka K., Earl H.J., Raizada M.N., Navabi A. (2020). A physio-morphological trait-based approach for breeding drought tolerant wheat. Front. Plant Sci..

[234] Araus J.L., Sanchez C., Edmeades G.O., Monneveux P., Ribaut J.M. (2011). Phenotyping maize for adaptation to drought. Drought Phenotyping in Crops: From Theory to Practice CGIAR Generation Challenge Program.

[235] Sinclair T.R. (2011). Challenges in breeding for yield increase for drought. Trends Plant Sci..

[236] Sinclair T.R., Purcell L.C., Sneller C.H. (2004). Crop transformation and the challenge to increase yield potential. Trends Plant Sci..

[237] Panguluri S.K., Kumar A.A. (2016). Phenotyping for Plant Breeding.

[238] Mir R.R., Reynolds M., Pinto F., Khan M.A., Bhat M.A. (2019). High-throughput phenotyping for crop improvement in the genomics era. Plant Sci..

[239] Hussain S., Mubeen M., Ahmad A., Akram W., Hammad H.M., Ali M., Masood N., Amin A., Farid H.U., Sultana S.R. (2019). Using GIS tools to detect the land use/land cover changes during forty years in Lodhran district of Pakistan. Environ. Sci. Pollut. Res..

[240] Fahlgren N., Gehan M.A., Baxter I. (2015). Lights, camera, action: High-throughput plant phenotyping is ready for a close-up. Curr. Opin. Plant. Biol..

[241] Barker III J., Zhang N., Sharon J., Steeves R., Wang X., Wei Y., Poland J. (2016). Development of a field-based high-throughput mobile phenotyping platform. Comput. Electron. Agric..

[242] Li L., Zhang Q., Huang D. (2014). A Review of Imaging Techniques for Plant Phenotyping. Sensors.

[243] Badigannavar A., Teme N., de Oliveira A.C., Li G., Vaksmann M., Viana V.E., Ganapathi T.R., Sarsu F. (2018). Physiological, genetic and molecular basis of drought resilience in sorghum [*Sorghum bicolor* (L.) Moench]. Ind. J. Plant Physiol..

[244] Fischer K.S., Fukai S., Kumar A., Leung H., Jongdee B., Monneveux P., Ribaut J.M. (2011). Phenotyping rice for adaptation to drought. Drought Phenotyping in Crops: From Theory to Practice: CGIAR Generation Challenge Program.

[245] Monneveux P., Jing R., Misra S.C. (2012). Phenotyping wheat for adaptation to drought using physiological traits. Front. Physiol..

[246] Passioura J.B. (2012). Phenotyping for drought tolerance in grain crops: When is it useful to breeders?. Funct. Plant Biol..

[247] Wang D., Fahad S., Saud S., Kamran M., Khan A., Khan M.N., Hammad H.M., Nasim W. (2019). Morphological acclimation to agronomic manipulation in leaf dispersion and orientation to promote “Ideotype” breeding: Evidence from 3D visual modeling of “super” rice (*Oryza sativa* L.). Plant Physiol. Biochem..

[248] Mutka A.M., Bart R.S. (2015). Image-based phenotyping of plant disease symptoms. Front. Plant Sci..

[249] Tardieu F., Cabrera-Bosquet L., Pridmore T., Bennett M. (2017). Plant phenomics, from sensors to knowledge. Curr. Biol..

[250] Singh B., Mishra S., Bohra A., Joshi R., Siddique K.H., Wani S.H. (2018). Crop phenomics for abiotic stress tolerance in crop plants. Biochemical, Physiological and Molecular Avenues for Combating Abiotic Stress Tolerance in Plants.

[251] Liu X., Hao L., Li D., Zhu L., Hu S. (2015). Long non-coding RNAs and their biological roles in plants. Genom. Proteom. Bioinf..

[252] Huanca-Mamani W., Arias-Carrasco R., Cárdenas-Ninasivincha S., Rojas-Herrera M., Sepúlveda-Hermosilla G., Caris-Maldonado J.C., Bastías E., Maracaja-Coutinho V. (2018). Long non-coding RNAs responsive to salt and boron stress in the hyper-arid Lluteno maize from Atacama Desert. Genes.

[253] Yu Y., Zhang Y., Chen X., Chen Y. (2019). Plant noncoding RNAs: Hidden players in development and stress responses. Annu. Rev. Cell Dev. Biol..

[254] Wang J., Meng X., Dobrovolskaya O.B., Orlov Y.L., Chen M. (2017). Non-coding RNAs and their roles in stress response in plants. Genom. Proteom. Bioinf..

[255] Dinger M.E., Pang K.C., Mercer T.R., Crowe M.L., Grimmond S.M., Mattick J.S. (2009). NRED: A database of long noncoding RNA expression. Nucleic Acids Res..

[256] Jha U.C., Nayyar H., Jha R., Khurshid M., Zhou M., Mantri N., Siddique K.H. (2020). Long non-coding RNAs: Emerging players regulating plant abiotic stress response and adaptation. BMC Plant Biol..

[257] Megha S., Basu U., Rahman M.H., Kav N.N. (2015). The role of long non-coding RNAs in abiotic stress tolerance in plants. Elucidation of Abiotic Stress Signaling in Plants.

[258] Wierzbicki A.T., Haag J.R., Pikaard C.S. (2008). Noncoding transcription by RNA polymerase Pol IVb/Pol V mediates transcriptional silencing of overlapping and adjacent genes. Cell.

[259] Li L., Eichten S.R., Shimizu R., Petsch K., Yeh C.T., Wu W., Chettoor A.M., Givan S.A., Cole R.A., Fowler J.E. (2014). Genome-wide discovery and characterization of maize long non-coding RNAs. Genome Biol..

[260] Di C., Yuan J., Wu Y., Li J., Lin H., Hu L., Zhang T., Qi Y., Gerstein M.B., Guo Y. (2014). Characterization of stress-responsive lncRNAs in Arabidopsis Thaliana by Integrating Expression, Epigenetic and Structural Features. Plant J..

[261] Zhang M., Zhao H., Xie S., Chen J., Xu Y., Wang K., Zhao H., Guan H., Hu X., Jiao Y. (2011). Extensive, clustered parental imprinting of protein-coding and noncoding RNAs in developing maize endosperm. Proc. Natl. Acad. Sci. USA.

[262] Chekanova J.A. (2015). Long non-coding RNAs and their functions in plants. Curr. Opin. Plant Biol..

[263] Böhmdorfer G., Wierzbicki A.T. (2015). Control of chromatin structure by long noncoding RNA. Trends Cell Biol..

[264] Zhang W., Han Z., Guo Q., Liu Y., Zheng Y., Wu F., Jin W. (2014). Identification of maize long non-coding RNAs responsive to drought stress. PLoS ONE..

[265] Amaral P.P., Dinger M.E., Mattick J.S. (2013). Non-coding RNAs in homeostasis, disease and stress responses: An evolutionary perspective. Brief. Funct. Genom..

[266] Li J.R., Liu C.C., Sun C.H., Chen Y.T. (2018). Plant stress RNA-seq nexus: A stress-specific transcriptome database in plant cells. BMC Genom..

[267] Pang J., Zhang X., Ma X., Zhao J. (2019). Spatio-temporal transcriptional dynamics of maize long non-coding RNAs responsive to drought stress. Genes.

[268] Qi X., Xie S., Liu Y., Yi F., Yu J. (2013). Genome-wide annotation of genes and noncoding RNAs of foxtail millet in response to simulated drought stress by deep sequencing. Plant Mol. Biol..

[269] Chung P.J., Jung H., Jeong D.H., Ha S.H., Choi Y.D., Kim J.K. (2016). Transcriptome profiling of drought responsive noncoding RNAs and their target genes in rice. BMC Genom..

[270] Xin M., Wang Y., Yao Y., Song N., Hu Z., Qin D., Xie C., Peng H., Ni Z., Sun Q. (2011). Identification and characterization of wheat long non-protein coding RNAs responsive to powdery mildew infection and heat stress by using microarray analysis and SBS sequencing. BMC Plant Biol..

[271] Budak H., Kaya S.B., Cagirici H.B. (2020). Long non-coding RNA in plants in the era of reference sequences. Front. Plant Sci..

[272] Wani S.H., Kumar V., Khare T., Tripathi P., Shah T., Ramakrishna C., Aglawe S., Mangrauthia S.K. (2020). miRNA applications for engineering abiotic stress tolerance in plants. Biologia.

[273] Tao Y., Zhao X., Mace E., Henry R., Jordan D. (2019). Exploring and Exploiting Pan-genomics for Crop Improvement. Mol. Plant.

[274] Edwards D., Batley J. (2016). Plant Genomics and Climate Change||The Impact of Genomics Technology on Adapting Plants to Climate Change. Plant Genom. Clim. Chang..

[275] Feuk L., Carson A.R., Scherer S.W. (2006). Structural variation in the human genome. Nat. Rev. Genet..

[276] Sebat J., Lakshmi B., Troge J., Alexander J., Young J., Lundin P., Månér S., Massa H., Walker M., Chi M. (2004). Large-scale copy number polymorphism in the human genome. Science.

[277] Pinkel D., Segraves R., Sudar D., Clark S., Poole I., Kowbel D., Collins C., Kuo W.L., Chen C., Zhai Y. (1998). High resolution analysis of DNA copy number variation using comparative genomic hybridization to microarrays. Nat. Genet..

[278] Saxena R.K., Edwards D., Varshney R.K. (2014). Structural variations in plant genomes. Brief. Funct. Genom..

[279] Danilevicz M.F., Fernandez C.G.T., Marsh J.I., Bayer P.E., Edwards D. (2020). Plant pangenomics: Approaches, applications and advancements. Curr. Opin. Plant Biol..

[280] Tranchant-Dubreuil C., Rouard M., Sabot F. (2018). Plant pangenome: Impacts on phenotypes and evolution. Annu. Plant Rev. Online.

[281] Tettelin H., Masignani V., Cieslewicz M.J., Donati C., Medini D., Ward N.L., Angiuoli S.V., Crabtree J., Jones A.L., Durkin A.S. (2005). Genome analysis of multiple pathogenic isolates of Streptococcus agalactiae: Implications for the microbial “pan-genome”. Proc. Natl. Acad. Sci. USA.

[282] Hirsch C.N., Foerster J.M., Johnson J.M., Sekhon R.S., Muttoni G., Vaillancourt B., Peñagaricano F., Lindquist E., Pedraza M.A., Barry K. (2014). Insights into the maize pan-genome and pan-transcriptome. Plant Cell.

[283] Schatz M.C., Maron L.G., Stein J.C., Wences A.H., Gurtowski J., Biggers E., Lee H., Kramer M., Antoniou E., Ghiban E. (2014). Whole genome de novo assemblies of three divergent strains of rice, *Oryza sativa*, document novel gene space of aus and indica. Genome Biol..

[284] Zhao Q., Feng Q., Lu H., Li Y., Wang A., Tian Q., Zhan Q., Lu Y., Zhang L., Huang T. (2018). Pan-genome analysis highlights the extent of genomic variation in cultivated and wild rice. Nat. Genet..

[285] Wang W., Mauleon R., Hu Z., Chebotarov D., Tai S., Wu Z., Li M., Zheng T., Fuentes R.R., Zhang F. (2018). Genomic variation in 3,010 diverse accessions of Asian cultivated rice. Nature.

[286] Montenegro J.D. (2017). The pangenome of hexaploid bread wheat. Plant J..

[287] Song J.M., Guan Z., Hu J., Guo C., Yang Z., Wang S., Liu D., Wang B., Lu S., Zhou R. (2020). Eight high-quality genomes reveal pan-genome architecture and ecotype differentiation of Brassica napus. Nat. Plants.

[288] Golicz A.A., Batley J., Edwards D. (2016). Towards plant pangenomics. Plant Biotechnol. J..

[289] Coletta R.D., Qiu Y., Ou S., Hufford M.B., Hirsch C.N. (2021). How the pan-genome is changing crop genomics and improvement. Genome Biol..

[290] Computational Pan-Genomics Consortium (2018). Computational pan-genomics: Status, promises and challenges. Brief Bioinform..

[291] Zuo W., Chao Q., Zhang N., Ye J., Tan G., Li B., Xing Y., Zhang B., Liu H., Fengler K.A. (2015). A maize wall-associated kinase confers quantitative resistance to head smut. Nat. Genet..

[292] Gamuyao R., Chin J.H., Pariasca-Tanaka J., Pesaresi P., Catausan S., Dalid C., Slamet-Loedin I., Tecson-Mendoza E.M., Wissuwa M., Heuer S. (2012). The protein kinase Pstol1 from traditional rice confers tolerance of phosphorus deficiency. Nature.

[293] Makalowski W., Gotea V., Pande A., Makalowski I., Anisimova M. (2019). Transposable elements: Classification, identification, and their use as a tool for comparative genomics. Evolutionary Genomics Methods in Molecular Biology.

[294] Wicker T., Sabot F., Hua-Van A., Bennetzen J.L., Capy P., Chalhoub B., Flavell A., Leroy P., Morgante M., Panaud O. (2017). A unified classification system for eukaryotic transposable elements. Nat. Rev. Genet..

[295] Dubin M.J., Scheid O.M., Becker C. (2018). Transposons: A blessing curse. Curr. Opin. Plant Biol..

[296] Gaut B.S., d’Ennequin M.L.T., Peek A.S., Sawkins M.C. (2000). Maize as a model for the evolution of plant nuclear genomes. Proc. Natl. Acad. Sci. USA.

[297] Elliott T.A., Gregory T.R. (2015). What’s in a genome? The C-value enigma and the evolution of eukaryotic genome content. Philos. Trans. R. Soc. Lond. B Biol. Sci..

[298] Lönnig W.E., Saedler H. (2002). Chromosome rearrangements and transposable elements. Annu. Rev. Genet..

[299] Zhang J., Yu C., Krishnaswamy L., Peterson T. (2011). Transposable elements as catalysts for chromosome rearrangements. Methods Mol. Biol..

[300] Jiang N., Ferguson A.A., Slotkin R.K., Lisch D. (2011). Pack-Mutator-like transposable elements (Pack-MULEs) induce directional modification of genes through biased insertion and DNA acquisition. Proc. Natl. Acad. Sci. USA.

[301] Fedoroff N.V. (2012). Transposable elements, epigenetics, and genome evolution. Science.

[302] Zhao D., Ferguson A.A., Jiang N. (2016). What makes up plant genomes: The vanishing line between transposable elements and genes. Biochim. Biophys. Acta.

[303] Ariel F.D., Manavella P.A. (2021). When junk DNA turns functional: Transposon-derived noncoding RNAs in plants. J. Exp. Bot..

[304] Lisch D. (2013). How important are transposons for plant evolution?. Nat. Rev. Genet..

[305] Anderson S.N., Stitzer M.C., Brohammer A.B., Zhou P., Noshay J.M., O’Connor C.H., Hirsch C.D., Ross-Ibarra J., Hirsch C.N., Springer N.M. (2019). Transposable Elements Contribute to Dynamic Genome Content in Maize. Plant J..

[306] Makarevitch I., Waters A.J., West P.T., Stitzer M., Hirsch C.N., Ross-Ibarra J., Springer N.M. (2015). Transposable elements contribute to activation of maize genes in response to abiotic stress. PLoS Genet..

[307] Yokosho K., Yamaji N., Fujii-Kashino M., Ma J.F. (2016). Retrotransposon-mediated aluminum tolerance through enhanced expression of the citrate transporter OsFRDL4. Plant Physiol..

[308] Wang H., Cimen E., Singh N., Buckler E. (2020). Deep learning for plant genomics and crop improvement. Curr. Opin. Plant Biol..

[309] Singh A., Ganapathysubramanian B., Singh A.K., Sarkar S. (2016). Machine learning for high-throughput stress phenotyping in plants. Trends Plant Sci..

[310] Hu H., Scheben A., Edwards D. (2018). Advances in integrating genomics and bioinformatics in the plant breeding pipeline. Agriculture.

[311] Brownlee J. (2016). Supervised and unsupervised machine learning algorithms. Mach. Learn. Mastery.

[312] Mahood E.H., Kruse L.H., Moghe G.D. (2020). Machine learning: A powerful tool for gene function prediction in plants. Appl. Plant Sci..

[313] Mejía-Guerra M.K., Buckler E.S. (2019). A k-mer grammar analysis to uncover maize regulatory architecture. BMC Plant Biol..

[314] Li H., Yin Z., Manley P., Burken J., Shakoor G., Fahlgren N., Mockler T. (2018). Early drought plant stress detection with bi-directional long-term memory networks. Photogramm. Eng. Remote. Sens..

[315] Ghosal S., Blystone D., Singh A.K., Ganapathysubramanian B., Singh A., Sarkar S. (2018). An explainable deep machine vision framework for plant stress phenotyping. Proc. Natl. Acad. Sci. USA.

[316] Wu B., Zhang H., Lin L., Wang H., Gao Y., Zhao L., Chen Y.P.P., Chen R., Gu L. (2019). A similarity searching system for biological phenotype images using deep convolutional encoder-decoder architecture. Curr. Bioinform..

[317] Libbrecht M.W., Noble W.S. (2015). Machine learning applications in genetics and genomics. Nat. Rev. Genet..

[318] Esposito S., Carputo D., Cardi T., Tripodi P. (2020). Applications and trends of machine learning in genomics and phenomics for next-generation breeding. Plants.

[319] Ma C., Zhang H.H., Wang X. (2014). Machine learning for big data analytics in plants. Trends Plant Sci..

[320] Xu C., Jackson S.A. (2019). Machine learning and complex biological data. Genome Biol..

[321] Kwon M.S., Lee B.T., Lee S.Y., Kim H.U. (2020). Modeling regulatory networks using machine learning for systems metabolic engineering. Curr. Opin. Biotechnol..

[322] Ni Y., Aghamirzaie D., Elmarakeby H., Collakova E., Li S., Grene R., Heath L.S. (2016). A machine learning approach to predict gene regulatory networks in seed development in Arabidopsis. Front. Plant Sci..

[323] Korani W., Clevenger J.P., Chu Y., Ozias-Akins P. (2019). Machine learning as an effective method for identifying true single nucleotide polymorphisms in polyploid plants. Plant Genome.

[324] Zhao J., Bodner G., Rewald B. (2016). Phenotyping: Using machine learning for improved pairwise genotype classification based on root traits. Front. Plant Sci..

[325] Selvaraj M.G., Manuel V., Diego G., Milton V., Henry R., Animesh A. (2020). Machine learning for high-throughput field phenotyping and image processing provides insight into the association of above and below-ground traits in cassava (*Manihot esculenta* Crantz). Plant Methods.

[326] Long P., Zhang L., Huang B., Chen Q., Liu H. (2020). Integrating genome sequence and structural data for statistical learning to predict transcription factor binding sites. Nucleic Acids Res..

[327] Sun L., Liu H., Zhang L., Meng J. (2015). lncRScan-SVM: A tool for predicting long non-coding RNAs using support vector machine. PLoS ONE.

[328] Gao X., Zhang J., Wei Z., Hakonarson H. (2018). DeepPolyA: A convolutional neural network approach for polyadenylation site prediction. IEEE Access.

[329] Ernst J., Kellis M. (2017). Chromatin-state discovery and genome annotation with ChromHMM. Nat. Protoc..

[330] Li Y., Shi W., Wasserman W.W. (2018). Genome-wide prediction of cis-regulatory regions using supervised deep learning methods. BMC Bioinform..

[331] Crane-Droesch A. (2018). Machine learning methods for crop yield prediction and climate change impact assessment in agriculture. Environ. Res. Lett..

